# Decoding Skeletal Biology Through Transcriptomics: Insights from Bulk, Single-Cell, Spatial, and Multi-Omics Approaches

**DOI:** 10.3390/ijms27188404

**Published:** 2026-09-21

**Authors:** Zayana Ali, Ahmad M. Alqudah, Lama Soubra, Chiara Cugno, Md Mizanur Rahman

**Affiliations:** 1 Biological Science Program, Department of Biological and Environmental Sciences, College of Arts and Sciences, Qatar University, Doha P.O. Box 2713, Qatar; za2211104@qu.edu.qa (Z.A.); aalqudah@qu.edu.qa (A.M.A.); 2 Environmental Science Program, Department of Biological and Environmental Sciences and Center of Sustainable Development, College of Arts and Sciences, Qatar University, Doha P.O. Box 2713, Qatar; lama.soubra@qu.edu.qa; 3 Advanced Cell Therapy Core, Research Department, Sidra Medicine, Doha P.O. Box 26999, Qatar; ccugno@sidra.org

**Keywords:** transcriptomics, RNA sequencing (RNA-seq), bulk RNA-seq, scRNA-seq, spatial transcriptomics, multi-omics, bone, BMSCs, osteoblast, osteoclast, bone diseases, biomarker discovery, precision medicine

## Abstract

Transcriptomic technologies have revolutionized our understanding of skeletal biology by shifting research from descriptive cellular characterization to a systems-level analysis of bone homeostasis and pathology. The rapid evolution of bulk RNA sequencing (bulk RNA-seq), single-cell RNA sequencing (scRNA-seq), spatial transcriptomics, and integrative multi-omics approaches has enabled unprecedented resolution of the molecular and cellular complexity of the skeletal microenvironment. Bone remodeling is a tightly regulated process driven by coordinated interactions among bone marrow-derived mesenchymal stem/stromal cells (BMSCs), osteoblasts, osteoclasts, osteocytes, immune cells, and other bone microenvironment components. This narrative review summarizes recent advances in bulk RNA-seq, scRNA-seq, spatial transcriptomics, and emerging multi-omics approaches that have transformed the study of bone development, remodeling, and disease. We discuss how transcriptomic analyses have revealed the heterogeneity of BMSCs and osteoblasts, elucidated the molecular mechanisms regulating osteoclast differentiation, and identified transcriptional changes associated with osteoclast dysregulation in metabolic, inflammatory, and age-related bone disorders. We further evaluate the limitations of bulk and scRNA-seq, including technical biases, loss of spatial information, and computational challenges. Finally, we highlight how spatial transcriptomics and integrative multi-omics approaches are overcoming these limitations by combining transcriptional, spatial, epigenetic, proteomic, and metabolomic data to provide a comprehensive understanding of skeletal biology, accelerate biomarker discovery, identify novel therapeutic targets, and advance precision medicine for bone diseases.

## 1. Introduction

Bone is a specialized connective tissue that provides structural support, protects vital organs, and serves as a metabolically active organ involved in mineral homeostasis [1]. In addition to its mechanical role, bone possesses a highly organized, porous architecture that accommodates the bone marrow, which contains diverse stem and progenitor cell populations essential for skeletal maintenance and blood-cell production [2,3]. Within the bone marrow cavity, two types of stem cells coexist: hematopoietic stem and progenitor cells (HSPCs), which sustain blood-cell production, and mesenchymal stromal/stem cells (MSCs), which contribute to skeletal lineages and provide essential support to the hematopoietic niche [4]. The coordinated interaction between these mesenchymal and hematopoietic compartments is essential for maintaining bone remodeling, hematopoietic function, and overall skeletal homeostasis [4].

Bone marrow-derived mesenchymal stem/stromal cells (BMSCs) are heterogeneous stromal populations derived from the bone marrow, with the capacity to differentiate toward multiple cell lineages, including adipocytes, osteoblasts, and chondrocytes [5]. These cells play essential roles in supporting skeletal development, maintaining the structural integrity of the bone marrow niche, and contributing to bone formation [6,7]. Osteogenic differentiation generates osteoblasts that produce bone matrix, while chondrogenic differentiation produces chondrocytes involved in cartilage formation and endochondral ossification. Adipogenic differentiation gives rise to marrow adipocytes. The balance among these lineages is essential for skeletal development, remodeling, and homeostasis [8]. BMSC lineage commitment is influenced by a combination of biological signals and physical and chemical cues within the cellular microenvironment. Alterations in these regulatory conditions can shift BMSC fate toward different lineages. In osteoporosis, a preferential bias toward adipogenic rather than osteogenic differentiation has been associated with reduced bone formation and increased marrow adiposity [9].

The phenotypic characteristics of BMSCs differ between native bone marrow populations and culture-expanded cells [10]. Culture-expanded BMSCs are commonly characterized by expression of CD73, CD90, and CD105, together with the absence of CD45, CD34, CD14/CD11b, CD19/CD79α, and HLA-DR [11]. Native BMSC populations have been identified using different combinations of positive and negative phenotypic markers, including CD45^low^/D7FIB^+^/CD271^+^ [12], CD64^bright^/CD31^bright^/CD14^−^ [13], Lin^−^/CD271^+^/CD45^−^/CD146^+^ [14], CD145^−^/CD34^−^/CD146^+^ [15,16], CD13^high^/CD105^+^/CD45^−^ [17] and PDPN^+^/CD146^−^/CD73^+^/CD164^+^ [18].

In addition to MSC populations, the bone marrow contains HSPCs, which self-renew and generate multiple blood lineages, including myeloid progenitors that give rise to osteoclasts [19,20,21]. Long-term hematopoietic stem cells (LT-HSCs) maintain long-term self-renewal and multilineage hematopoietic potential, whereas short-term HSCs (ST-HSCs) have more restricted self-renewal capacity and primarily support short-term hematopoietic reconstitution [22]. ST-HSCs subsequently generate multipotent progenitors (MPPs), which retain broad differentiation potential but have limited self-renewal capacity. Further differentiation produces lineage-specific progenitors, including common myeloid progenitors (CMPs) and common lymphoid progenitors (CLPs). CMPs give rise to granulocyte–macrophage progenitors (GMPs) and megakaryocyte–erythroid progenitors (MEPs), while CLPs generate lymphoid lineages, including B, T, and natural killer (NK) cells [23,24]. Within the myeloid compartment, monocytes derived from bone marrow myeloid progenitors circulate in the peripheral blood and can subsequently migrate into tissues, where they contribute to macrophage populations and specialized cell types, including osteoclasts. Thus, osteoclasts arise from the hematopoietic myeloid lineage and share developmental origins with monocytes and macrophages [25,26,27]. Together, these mesenchymal and hematopoietic lineages generate the principal bone-forming and bone-resorbing cell populations that collectively maintain skeletal homeostasis.

**Figure 1:**
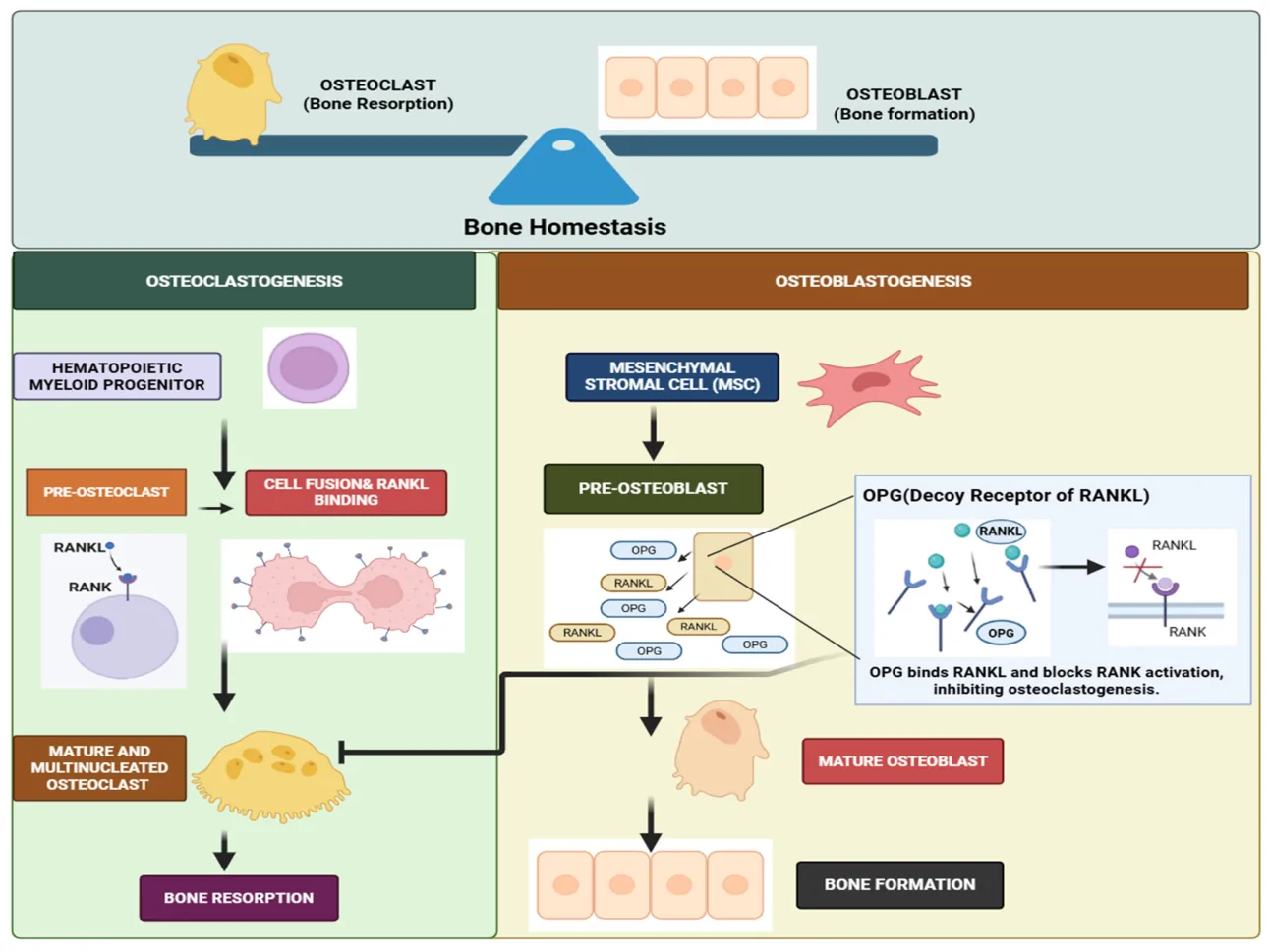
Schematic representation of the bone remodeling cycle: Bone remodeling relies on the balanced activity of osteoclasts (bone resorption) and osteoblasts (bone formation). Osteoclastogenesis is driven by RANKL-RANK signaling and negatively regulated by osteoprotegerin (OPG), which acts as a decoy receptor to limit resorption and maintain skeletal homeostasis.

Given bone’s critical role in the human body, skeletal tissue must be continuously renewed to maintain strength and structural integrity. This renewal occurs through bone remodeling, a dynamic process involving two key mechanisms: bone matrix formation and bone resorption [28]. Osteoblasts, derived from BMSCs, form new bone matrix, whereas osteoclasts, derived from HSPC-derived myeloid progenitors, resorb old or damaged bone [29]. The coordinated activity of osteoblasts and osteoclasts is essential for maintaining bone homeostasis [1] (Figure 1), and disruption of this equilibrium leads to metabolic bone disorders [30]. Understanding the cellular and molecular mechanisms that regulate bone remodeling is therefore essential for elucidating the pathogenesis of skeletal disorders and identifying potential therapeutic targets.

Traditional approaches for studying bone cell populations, including imaging techniques, flow cytometry, and lineage-tracing models, have provided important insights into skeletal biology but are inherently constrained by their reliance on predefined markers, which often lack cell-type specificity and are shared across multiple cellular populations [31]. Many of these methods overlook rare or transitional cell states and provide only a partial representation of the underlying molecular complexity within bone tissue [31]. Consequently, there is a growing need for more comprehensive approaches that can resolve cellular diversity at higher resolution.

Transcriptomic profiling has emerged as a powerful strategy to address these limitations by enabling the analysis of gene expression patterns at the RNA level [32]. The transcriptome, the complete set of RNA molecules in a cell, provides a dynamic framework for studying bone biology by revealing cellular heterogeneity, distinct bone cell populations, and molecular programs that regulate osteoblast and osteoclast development. RNA sequencing has further advanced transcriptomic research by enabling comprehensive, genome-wide profiling of gene expression [33].

RNA sequencing can be performed at both bulk tissue and single-cell levels. While bulk RNA sequencing (bulk RNA-seq) provides a comprehensive overview of gene expression across a tissue sample, its reliance on averaged signals obscures cellular heterogeneity and limits the resolution of rare or functionally distinct cell populations [34,35]. In contrast, single-cell RNA sequencing (scRNA-seq) enables transcriptomic analysis at the level of individual cells, allowing for the identification of cellular heterogeneity, rare subpopulations, and dynamic differentiation trajectories [35,36,37,38,39]. Unlike traditional approaches such as flow cytometry, magnetic-activated cell sorting (MACS), immunohistochemistry (IHC), and in situ hybridization (ISH), which assess a limited number of selected markers, scRNA-seq enables comprehensive transcriptome profiling at single-cell resolution with greater sensitivity and accuracy [40]. This high-resolution approach has been instrumental in uncovering previously unrecognized diversity within bone marrow cell populations and in defining the molecular programs that regulate osteoblast and osteoclast differentiation.

scRNA-seq alone does not capture spatial context or higher-order tissue architecture. In this regard, spatial transcriptomics has emerged as a complementary approach that enables gene expression profiling while preserving spatial localization within intact tissue sections [41]. Integration of scRNA-seq with spatial transcriptomic data allows precise mapping of cell populations within their native microenvironment, thereby improving our knowledge of tissue organization and cell–cell interactions in bone [42]. Furthermore, multi-omics strategies that integrate transcriptomic, epigenomic, proteomic, and metabolomic datasets have emerged, enabling a more comprehensive view of the regulatory networks governing skeletal biology [43]. Collectively, these integrative approaches are transforming bone research from single-dimensional gene expression analysis to a systems-level understanding of skeletal development and disease.

Several recent reviews have offered valuable perspectives on applying transcriptomic and multi-omics technologies in skeletal biology. However, individual studies frequently focus on either the bone-forming or bone-resorbing lineage in isolation. Furthermore, many investigate differentiation using a single transcriptomic platform without providing a comprehensive, stage-by-stage synthesis of the transcriptional programs that govern progression from progenitor populations to mature osteoblasts or osteoclasts. To address this gap, this review integrates transcriptomic evidence across bulk, single-cell, spatial, and multi-omics platforms to reconstruct differentiation trajectories in both osteoblast and osteoclast lineages, alongside detailed profiling of BMSCs. A central focus is the stage-resolved organization of osteoblast and osteoclast differentiation, extending from early progenitors and precursor populations through intermediate states to mature functional cells. Rather than focusing solely on individual transcriptomic technologies or cellular populations, this review integrates findings from multiple transcriptomic studies to compile genes, transcription factors, and regulatory signatures associated with each major developmental stage and organize them by reported cellular state and functional relevance. Additionally, the accompanying tables integrate cross-study findings, allowing direct comparison of stage-associated markers identified independently across datasets. The recurrent identification of specific gene sets across independent studies provides convergent evidence supporting their association with distinct cellular states, while discrepancies highlight transcriptional variation across experimental contexts. By distinguishing between human and animal evidence, accounting for variations in sample sources, and connecting normal differentiation trajectories to their disruption in skeletal pathology, this review provides a consolidated, stage- and lineage-oriented framework for interpreting transcriptomic signatures in bone health and disease.

## 2. Databases and Literature Search Strategy

This narrative review provides a comprehensive overview of the evolution and application of transcriptomic technologies in skeletal biology, with particular emphasis on their use in studying skeletal development, bone remodeling, cellular heterogeneity, osteoblast and osteoclast differentiation, and bone-related diseases. We conducted a structured literature search to identify relevant peer-reviewed studies using transcriptomic approaches, including bulk RNA-seq, scRNA-seq, spatial transcriptomics, and integrative multi-omics approaches.

The literature search was conducted using PubMed, Scopus, Web of Science, Science Direct, and Google Scholar. Searches were performed using combinations of keywords related to skeletal biology and transcriptomic technologies, including “bone biology,” “skeletal biology, “ BMSCs”, “ MSCs,” “HSCs,” “osteoblast,” “osteoclast,” “osteocyte,” “bone remodeling,” “transcriptomics,” “RNA sequencing,” “bulk RNA-seq,” “scRNA-seq,” “spatial transcriptomics,” and “multi-omics.” Boolean combinations included (“bone” AND “transcriptomics”), (“RNA sequencing” AND “bone”), (“scRNA-seq” AND “osteoblast”), (“scRNA-seq” AND “osteoclast”), (“BMSC” AND “RNA-seq”), (“spatial transcriptomics” AND “bone”), (“multi-omics” AND “skeletal biology”), and (“bone remodeling” AND “gene expression”). The search primarily included studies published between 2000 and 2026. We also considered landmark studies published before 2000 that provided foundational knowledge relevant to skeletal biology, bone-cell biology, or the development of transcriptomic approaches. We selected studies based on their relevance to the review’s objectives and scope. We prioritized original peer-reviewed research articles investigating transcriptomic technologies in bone cell populations, skeletal development, bone remodeling, and bone-related diseases. Studies were excluded if they were not directly related to transcriptomic analysis in skeletal biology, lacked sufficient methodological detail, or consisted of conference abstracts, editorials, unpublished reports, or other non-peer-reviewed literature. As this is a narrative review, a formal systematic quality assessment was not performed. However, we critically evaluated all included studies based on methodological details, experimental design, transcriptomic platform, sample type, and relevance to the topic to ensure a balanced, up-to-date synthesis of the current literature.

## 3. RNA Sequencing-Based Analysis of Bone Cells

### 3.1. Transcriptomic Landscape of BMSC Heterogeneity

**Figure 2:**
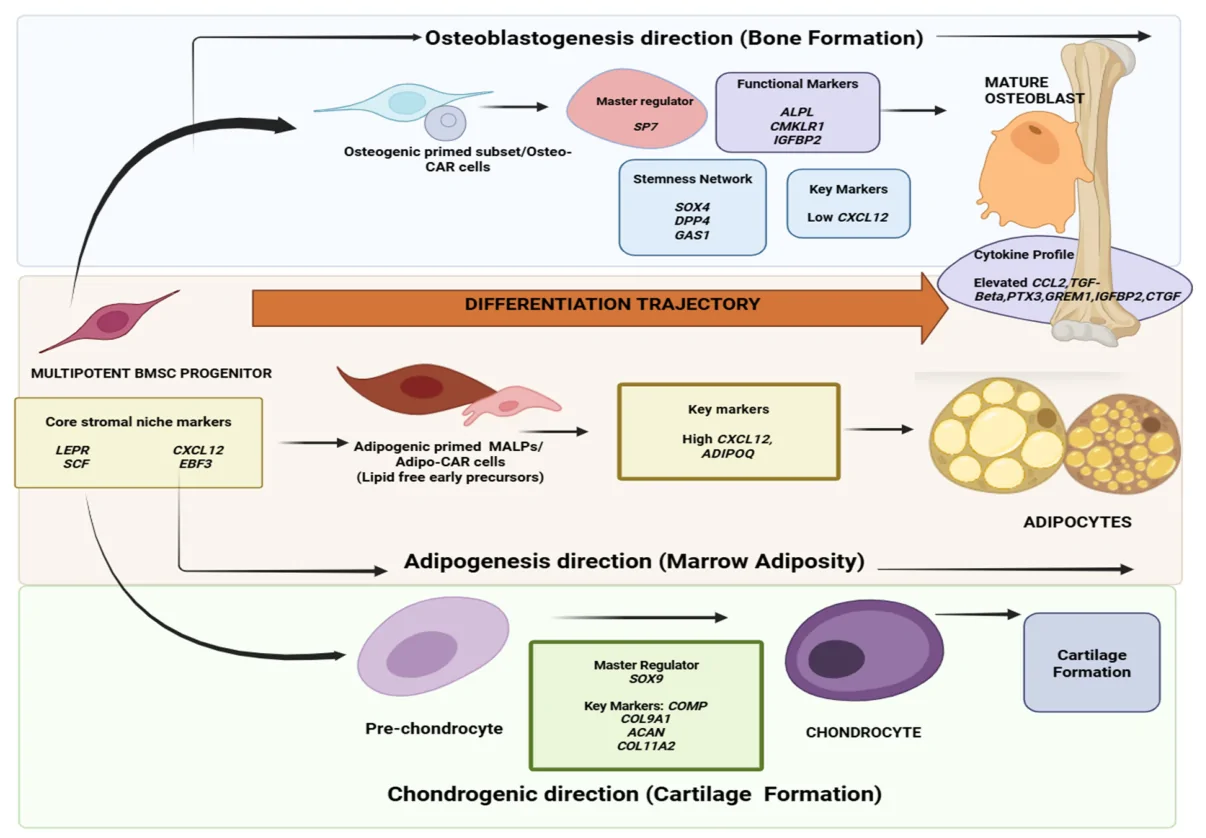
scRNA-seq derived differentiation trajectory of multipotent BMSCs: Schematic trajectory of multipotent BMSC progenitors differentiating into mature osteoblasts (bone formation), adipocytes (fat formation) and chondrocytes (cartilage formation), highlighting key cluster-specific marker genes, functional networks, and transcription factors identified via transcriptomic analysis.

BMSCs constitute a heterogeneous mesenchymal cell population that plays a central role in maintaining the skeleton and the bone marrow microenvironment. scRNA-seq has enabled high-resolution profiling of their transcriptional heterogeneity and distinct stromal cell populations (Figure 2), revealing distinct cellular subsets that were previously difficult to resolve with conventional approaches [44]. scRNA-seq analyses have demonstrated that many BMSCs exhibit a reticular morphology and express characteristic stromal markers, including C-X-C motif ligand 12 (*Cxcl12*) [45,46], leptin receptor (*Lepr*) [31,43,47], stem cell factor (*Scf*) [48], and early B-cell factor 3 (*Ebf3*) [49], which are closely associated with stromal niche function and skeletal progenitor activity. Reticular stromal cells constitute approximately 0.3% of the total bone marrow cellular compartment, and the Cxcl12^+^Lepr^+^ subset is predominantly localized within the marrow cavity and has been described as the BMSC population [46]. Early studies demonstrated substantial overlap between Lepr^+^ stromal cells and *Cxcl12*-abundant reticular (CAR) cells [50]. Subsequent scRNA-seq analyses revealed greater heterogeneity within this compartment, identifying multiple BMSC-like populations with high Lepr and Cxcl12 expression that share molecular features with previously described CAR cells. These stromal populations are important components of the hematopoietic niche, providing factors that support hematopoietic stem and progenitor cells [51]. These cells also contribute substantially to bone and adipocyte formation in adult bone marrow and regulate osteogenic and adipogenic differentiation [52,53].

Beyond defining stromal identity, transcriptomic analyses have revealed important insights into the regulatory programs that govern BMSC differentiation into osteogenic and adipogenic lineages [41]. scRNA-seq studies have identified distinct Lepr^+^Cxcl12^+^ MSC subpopulations with osteogenic or adipogenic transcriptional profiles [52,53,54]. The Lepr^+^Cxcl12^+^ MSC compartment comprises distinct lineage-primed populations, including adipogenic-primed cells characterized by high Cxcl12 and adipogenic marker Adipoq expression, and osteogenic-primed cells exhibiting relatively lower Cxcl12 expression and increased expression of osteogenic genes such as Sp7 and Alpl [55]. Within the Lepr^+^ compartment, a distinct adipogenic population characterized by Adipoq expression but lacking mature lipid accumulation has been identified as marrow adipogenic lineage precursors (MALPs).

scRNA-seq and pseudotime analyses have further supported this lineage heterogeneity, placing early BMSC-like mesenchymal progenitors upstream of distinct osteoblast and adipocyte populations, supporting the potential for lineage diversification toward either fate [53]. Consistently, complementary findings from murine models have further refined stromal cell classification, showing that CAR cells are enriched in multipotent stromal cells and progenitors of both adipocytes and osteoblasts, further supporting their diverse lineage potential [53,56]. Further analysis of Cxcl12^+^ BMSCs in *Cxcl12*-GFP mouse models has identified two principal CAR cell populations: preadipocyte-like reticular cells (Adipo-CAR) and preosteoblast-like cells (Osteo-CAR), representing lineage-primed stromal subsets [43]. scRNA-seq combined with trajectory analysis has further elucidated the hierarchical organization of Lepr^+^ BMSCs, revealing their skeletal stem-like properties, including self-renewal capacity and lineage progression. Studies using young adult and aging Lepr-Cre mouse models have demonstrated that Lepr-Cre labeling marks the majority of BMSCs and osteogenic lineage cells in adult long bones, while the labeled BMSC compartment also contains adipogenic populations, including Adipo-CAR cells and MALPs, indicating that Lepr^+^ stromal cells encompass both osteogenic and adipogenic populations [57].

Further insights into stromal cell heterogeneity have been provided by scRNA-seq studies of human BMSCs. Zhongyu Xie et al. identified multiple transcriptionally distinct subpopulations, including a stemness-associated cluster characterized by high expression of *SOX4*, *GAS1*, and *DPP4* [58]. This subpopulation is associated with maintaining stem cell properties and exhibits transcriptional features linked to adipogenic potential and skeletal development. In contrast, a differentiation-primed subpopulation exhibited elevated expression of genes encoding cytokines and osteogenic/adipogenic regulatory factors, including *CCL2*, *IGFBP2*, and *CMKLR1*, suggesting a shift toward osteogenic commitment and a potential role as a precursor population contributing to bone formation. Notably, the CMKLR1^+^ subpopulation displayed higher expression of cytokines and other stromal factors, including *CCL2*, *TGF-β*, *IGFBP2*, *PTX3*, *GREM1*, and *CTGF*, compared with the stemness-associated population [58]. Functional analyses further demonstrated that CMKLR1 promotes osteogenic differentiation while suppressing adipogenesis in BMSCs.

In summary, earlier studies suggested that several of the stromal populations identified by different markers might partially overlap, making their individual identities and functional significance difficult to distinguish. However, scRNA-seq has refined the view of BMSCs as a relatively uniform population by revealing substantial transcriptional heterogeneity within the Lepr^+^/Cxcl12^+^ compartment. In particular, CAR populations can be resolved into distinct osteogenic- and adipogenic-primed subsets, indicating that CAR cells do not represent a uniform population with equivalent differentiation potential. Within the broader Lepr^+^ BMSC compartment, distinct populations have also been associated with both osteogenic and adipogenic differentiation, supporting the contribution of Lepr^+^ stromal cells to both lineages. More broadly, scRNA-seq studies have identified several stromal states, including mesenchymal progenitors with osteogenic–chondrogenic potential, osteogenic or adipogenic progenitors, and cells with more committed osteogenic or adipogenic profiles [59]. Together, these findings suggest that the BMSC compartment comprises a continuum of multipotent and lineage-primed populations rather than a single homogeneous population with uniform differentiation potential.

### 3.2. Transcriptomic Insights into Osteoblast Heterogeneity

During bone regeneration, BMSC homing, osteogenic differentiation, extracellular matrix (ECM) synthesis and mineralization, and maturation into osteocytes are key steps in bone formation. Osteoblasts are bone-forming cells that account for approximately 4–6% of the cellular content within the bone lineage [60]. Their primary function is the synthesis and mineralization of the ECM, which is essential for bone formation and remodeling. These bone-forming cells originate from BMSC-derived progenitors that progressively commit to the osteogenic lineage under the control of specific transcriptional regulators [61]. Mature osteoblasts synthesize key ECM proteins, including type I collagen, which constitutes the majority of the organic bone matrix, along with markers such as osteocalcin and alkaline phosphatase [62]. Initially, this matrix is deposited in an unmineralized form known as osteoid, which subsequently undergoes mineralization through the deposition of calcium phosphate crystals, primarily as hydroxyapatite [63]. Osteoblasts are central to remodeling, as they are responsible for generating the organic components of the bone matrix and subsequently contributing to the osteocyte population [64]. During remodeling, a proportion of osteoblasts become embedded within the matrix and differentiate into osteocytes, while the remaining cells are eliminated through programmed cell death [64].

BMSCs residing within the marrow environment maintain their ability to differentiate into multiple cell types and give rise to both osteoblast and adipocyte lineages [65]. The balance between these fates is regulated by extracellular signaling inputs, soluble factors, and lineage-defining transcriptional regulators, most prominently *RUNX2* in osteogenesis and *PPARG* in adipogenesis [66]. Early osteogenic commitment is orchestrated by signaling pathways including Wnt/β-catenin, bone morphogenetic proteins (BMPs), and Hedgehog signaling [67]. These signaling cascades converge to establish stage-specific transcriptional programs that define lineage specification.

The dynamics of these gene expression changes have been extensively characterized using scRNA-seq analysis of human BMSCs. *RUNX2* and *OSTERIX (SP7)* function as sequential master regulators of osteogenic differentiation, driving progression toward the osteoblast lineage [68]. Downstream osteogenic genes, including alkaline phosphatase (*ALPL)*, osteoprotegerin *(TNFRSF11B* (OPG)), type I collagen (*COL1A1*), and osteocalcin (*BGLAP*), are expressed in a stage-dependent manner during osteoblast maturation. In contrast, adipogenic differentiation is regulated by transcription factors such as *PPARG* and CCAAT/enhancer-binding protein alpha. Concurrently, commitment toward the osteogenic lineage is accompanied by the active suppression of alternative differentiation programs, including adipogenic genes such as *PPARG* and *LEP*, and chondrogenic genes such as *COMP*, *COL9A1*, *COL11A2*, and *ACAN*, thereby ensuring the maintenance of osteogenic identity and lineage fidelity [64].

**Figure 3:**
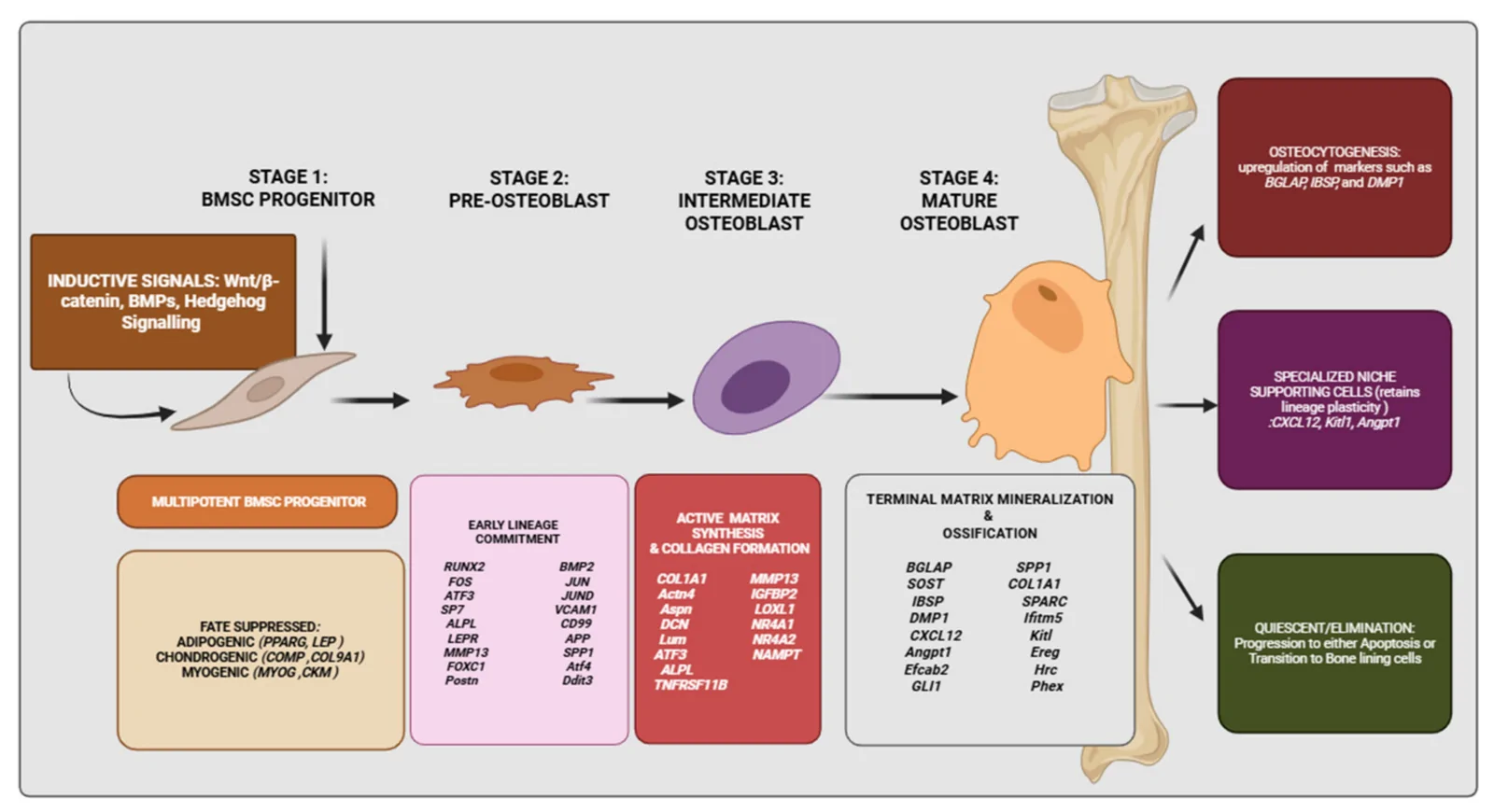
RNA-seq derived four-stage model of osteoblast differentiation and heterogeneity: Schematic illustration mapping the four successive transcriptional stages (progenitor, preosteoblast, intermediate, and mature) governing osteogenesis. The model integrates stage-specific marker gene expression networks and signature transcription factors with the divergent downstream functional fates (osteocytogenesis, niche support, or quiescence/apoptosis) that characterize mature osteoblast heterogeneity.

scRNA-seq, particularly when combined with pseudotime trajectory analysis, has provided detailed resolution of the transcriptional dynamics underlying osteogenic differentiation [69,70] (Figure 3). These analyses indicate that osteoblast differentiation originates from Lepr^+^/Cxcl12^+^ BMSC progenitor populations that initially express stromal and adipogenic-associated signatures. As cells progress toward a committed osteoblast state, adipogenic-associated genes such as ADIPOQ, LPL, and APOE become progressively less prominent, whereas osteogenic marker genes, including RUNX2, SP7, and ALPL, become increasingly enriched.

#### 3.2.1. Human Studies

A study using scRNA-seq of human bone marrow identified multipotent BMSC populations and several transcriptionally distinct stromal subsets with multilineage differentiation potential [71]. The multipotent stromal population expressed high levels of CXCL12, LEPR, DCN, and PTGDS, together with adipogenic genes (CEBPD, LPL, PLIN1, ADIPOQ, CCL2, and PPARG) and osteogenic-associated genes (GAS6, FBN1, ALPL, RUNX1, SPARCL1, and CDH11), consistent with both adipogenic and osteogenic potential. A distinct adipogenic population, termed highly adipocytic gene-expressing progenitors (HAGEPs), expressed adipogenic marker genes together with stress-responsive transcription factors FOS, FOSB, JUNB, and EGR1 and was identified as an adipocyte-progenitor population. Another stromal subset with higher expression of osteogenic-associated genes, including RUNX1, CDH11, EBF1, and EBF3, was characterized as preosteoblasts with an osteogenic tendency. In addition, a population enriched for osteochondrogenic markers, including BGLAP, CHAD, SPP1 (OPN), RUNX2, CDH11, CDH2, IBSP (BSP), and SPARC, was identified as osteochondrogenic progenitors. These findings show that human bone marrow stromal cells comprise transcriptionally distinct populations with varying degrees of lineage commitment rather than a uniform pool of multipotent cells.

Building on this broader characterization of human stromal populations, Liu et al. performed detailed scRNA-seq analysis of human BMSCs and osteoblast-lineage cells [72]. Eight transcriptionally distinct clusters (C1–C8) were identified based on highly variable genes, comprising three BMSC clusters (BMSC1–3), three preosteoblast clusters (PreOB1–3), and clusters representing immature and mature osteoblasts. Gene Ontology enrichment analysis revealed distinct molecular signatures across these populations, with most clusters expressing osteoblast-associated genes such as ALPL, COL1A1, and ITGB1. Within the BMSC compartment, BMSC2 displayed adipogenic characteristics, marked by elevated *ADIPOQ* expression, whereas BMSC3 lacked key osteogenic genes, including *ALPL* and *ITGB1*, and was interpreted as a terminal BMSC state with limited differentiation potential. ADAM28 (ADAM metallopeptidase domain 28), a member of the ADAM family of multifunctional cell-surface and secreted glycoproteins, was highly expressed in BMSC1 but nearly absent in BMSC2 and was associated with promoting osteogenic rather than adipogenic differentiation of BMSCs. The study further showed that preosteoblasts represented a transitional state between skeletal progenitors and mature osteoblasts, retaining BMSC-associated genes such as LEPR and THY1, while showing high expression of MGP and IGFBP4. MGP promoted bone formation through Wnt/β-catenin signaling, whereas IGFBP4 limited BMSC proliferation and thereby restrained excessive osteogenic differentiation. The study identified distinct preosteoblast states: PreOB2 showed high expression of NR4A1 and NR4A2, associated with osteoblast differentiation and inhibition of osteoclast recruitment, suggesting a role in osteoblast maturation. In contrast, the previously unreported PreOB3 cluster highly expressed ATF3, CCL2, CXCL2, IRF1, and IER3 and was associated with a less differentiated state and osteoclast recruitment. Pseudotime analysis positioned mature osteoblasts at the terminal end of the osteogenic trajectory, characterized by high expression of the late osteoblast markers IBSP, BGLAP, and SPP1. Mapping of osteogenic regulatory factors further supported the inferred trajectory, with IBSP enriched in mature osteoblasts, while early osteogenic genes such as LEPR and CXCL12 were absent or markedly reduced in mature osteoblasts. The study also found that FZD1 was highly expressed in BMSCs associated with the osteogenic differentiation trajectory, whereas WIF1 and SFRP4, which were enriched in mature osteoblasts, have been reported to inhibit osteogenic differentiation.

Further insight into the hierarchical transition from progenitors to mature cells was provided by Hui-Xi Zhang et al., who utilized scRNA-seq to characterize the transcriptional diversity within human primary osteoblast populations [73]. Their analysis identified nine transcriptionally distinct clusters representing hierarchical stages of osteoblast differentiation. Early osteoblast progenitors express markers such as *LEPR*, *FOXC1*, and AP-1 family genes (*FOS*, *JUN*, *JUNB*, *JUND*), which transition into preosteoblasts co-expressing mesenchymal markers (*LEPR*, *VCAM1*) alongside *CD99* and *APP*. Intermediate clusters exhibit stage-specific transcriptional programs, including *IGFBP2/LOXL1*, *NR4A1/NR4A2*, and *ATF3/NAMPT*, ultimately progressing to mature osteoblasts defined by high expression of mineralization-associated genes such as *BGLAP*, *SPP1*, *IBSP*, *RUNX2*, and *SP7* [73]. This stage-resolved analysis provides further evidence that human osteoblast differentiation is characterized by progressive changes in transcriptional programs across distinct cellular states.

Complementary evidence was provided by a transcriptomic study using 20 independent healthy human induced pluripotent stem cell (iPSC) lines to characterize osteogenic differentiation across three defined stages: MSC, preosteoblast and osteoblast [74]. Progressive osteogenic differentiation was accompanied by stage-specific changes in gene expression, including increased expression of osteogenic markers such as ALPL, COL1A1, RUNX2, SPARC, OMD, and OGN. The transcriptomic profiles of iPSC-derived MSCs and osteoblasts also resembled those of primary human MSCs and osteoblasts, respectively, supporting the relevance of this model to human osteogenesis. Comparative analysis identified extensive transcriptional remodeling between the MSC, preosteoblast, and osteoblast stages, including differential expression of 840 transcription factor genes. Among these, KLF16 was identified as a potential regulator of osteogenic differentiation. Functional validation demonstrated that KLF16 overexpression inhibited osteogenic differentiation and mineralization in vitro, while Klf16 haploinsufficiency in mice was associated with increased bone mineral density, trabecular number, and cortical bone area, providing in vivo support for its inhibitory role in osteogenesis. Thus, this study complements primary-tissue scRNA-seq findings by linking stage-specific transcriptional changes to functional regulation of osteogenesis.

Beyond differentiation trajectories, transcriptomic analyses have also revealed additional molecular features associated with mature human osteoblasts. Meshcheryakova et al. performed an integrative analysis of publicly available gene-expression datasets focusing on RNA-binding protein (RBP)-associated transcriptional patterns in osteoblasts [75]. The study identified 19 genes encoding RBPs that were highly expressed in osteoblast populations, including *U2SURP*, *NONO*, *LARP6*, *P3H1*, *FAM98B*, *HNRNPA3*, *RBFOX2*, *PRMT1*, *FIP1L1*, *SAFB*, *ERAL1*, *RBMS2*, *HNRNPA0*, *DDX1*, *SF3A3*, *ZC3H7A*, *YBX3*, *MATR3*, and *CPSF7*. In addition, several genes encoding RBPs associated with bone metabolism, including *IGF2BP3*, *FUS*, *LCORL*, *TRIM25*, *CPSF6*, *EPRS*, *IARS*, *ADARB1*, *FAM98A*, *UPF1*, *HNRNPA2B1*, and *ZC3H7B*, were significantly regulated following osteogenic stimulation with dexamethasone, β-glycerophosphate, BMP, and bisphosphonate. Dexamethasone predominantly increased the expression of these genes, whereas β-glycerophosphate predominantly decreased their expression, highlighting their responsiveness to osteogenic cues. Among these genes, *RBFOX2* showed high expression in osteoblasts, whereas *RBMS2* emerged as a potential novel regulator with limited prior association with bone biology. Table 1 summarizes the key transcriptomic markers, their functional roles, and the osteoblast differentiation stages identified across these human studies.

#### 3.2.2. Animal Studies

Transcriptomic studies in animal models have further characterized the cellular heterogeneity and regulatory programs underlying osteoblast differentiation. Longitudinal bulk RNA-seq profiling of murine primary calvarial osteoblasts by Layal Abo Khayal et al. provides temporal insight into osteoblast differentiation [76]. Early osteogenic induction was characterized by elevated expression of *Bmp2* and *Runx2*, together with stress-responsive transcription factors such as *Atf3*, *Atf4*, and *Ddit3*, indicating active lineage commitment. As differentiation progressed, the transcriptional program shifted toward an intermediate stage associated with ECM formation, characterized by increased expression of *Col1a1* and *Actn4*, as well as the small leucine-rich proteoglycans *Aspn*, *Dcn*, and *Lum*. Notably, *Col1a1* expression peaked during this phase, reflecting active matrix synthesis, whereas *Mmp13* displayed a biphasic expression pattern, with increased expression during both early and late phases of this intermediate stage [76]. Late maturation and mineralization were characterized by increased *Bglap2*, *Sost*, *Ibsp*, *Dmp1*, and *Spp1*, supporting progression toward terminal mineralization.

While bulk RNA-seq captures gene-expression changes across differentiation, scRNA-seq further resolves the cellular heterogeneity underlying these stages. Murine BMSC analysis by Baryawno et al. [70] and Chai et al. [55] provided further insight into murine osteoblast differentiation and heterogeneity. The studies demonstrate that preosteoblasts express key transcription factors such as *Runx2* and *Sp7*, alongside early osteogenic markers including *Mmp13*, *Postn*, and *Spp1*. At this preosteoblast stage, *Runx2* initiates osteogenic commitment, whereas *Sp7* reinforces differentiation and maturation [69], together activating gene programs governing type I collagen-based ECM formation [67], thereby supporting early osteoblastogenic activity. Intermediate osteoblast states showed variable expression of classical osteogenic markers including *Alpl*, *Tnfrsf11b* (OPG) and *Col1a1*, reflecting asynchronous maturation and functional specialization. In terminal differentiation stages, mature osteoblasts are characterized by high expression of mineralization and ECM genes, including *Bglap2*, *Col1a1*, *Sparc*, and *Ifitm5* [55,70]. However, scRNA-seq analyses further demonstrate that even mature osteoblasts comprise distinct sub-clusters with differential functional roles, including subsets specialized for matrix production, mineralization, and niche regulation, highlighting persistent transcriptional heterogeneity within the mature osteoblast compartment.

The heterogeneity observed among mature osteoblasts was further demonstrated by Hirotaka Yoshioka et al., who performed scRNA-seq on Col1a1-driven Venus^+^ osteoblasts isolated from neonatal murine calvariae and identified two distinct developmental trajectories [77]. One trajectory represents canonical maturation toward osteocytogenesis, characterized by *Bglap*, *Ibsp*, and *Dmp1*. The second diverged toward a population co-expressing osteoblast-associated genes (*Dmp1*, *Phex*) with progenitor- and niche-associated genes including *Cd34*, *Cxcl12*, *Kitl1*, and *Angpt1*. These findings suggest that some mature osteoblasts retain lineage-associated plasticity or acquire specialized niche-supporting functions rather than progressing exclusively toward terminal osteocytogenesis.

Beyond defining differentiation states, transcriptomic studies have also identified regulators of osteoblast maturation. Myungsuk Kim et al. used bulk RNA-seq to investigate the effects of phytoestrogen genistein on osteogenesis in mouse MC3T3-E1 osteoblastic cells [78]. Differential expression analysis revealed that genistein upregulates genes such as *Ereg* and *Efcab2*, which are critical for osteoblast maturation, as their depletion reduces *Alp* activity, ECM mineralization, and the expression of key osteogenic markers, including *Runx2*, *Alp*, and *Bmp2.* Conversely, *Hrc*, *Gli1*, and *Ifitm5* were downregulated, and their knockdown enhanced osteoblast maturation, suggesting inhibitory roles in differentiation. These findings show how transcriptomic profiling can extend beyond identifying differentiation-associated markers to identify potential positive and negative regulators of osteoblast maturation. Table 2 summarizes the key transcriptomic markers, their functional roles, and the osteoblast differentiation stages identified across these mouse models.

Collectively, human and animal studies support a progressive osteoblast differentiation trajectory from progenitor and preosteoblast states through intermediate stages to mature osteoblasts.

### 3.3. Transcriptomic Insights into Osteoclast Differentiation

**Figure 4:**
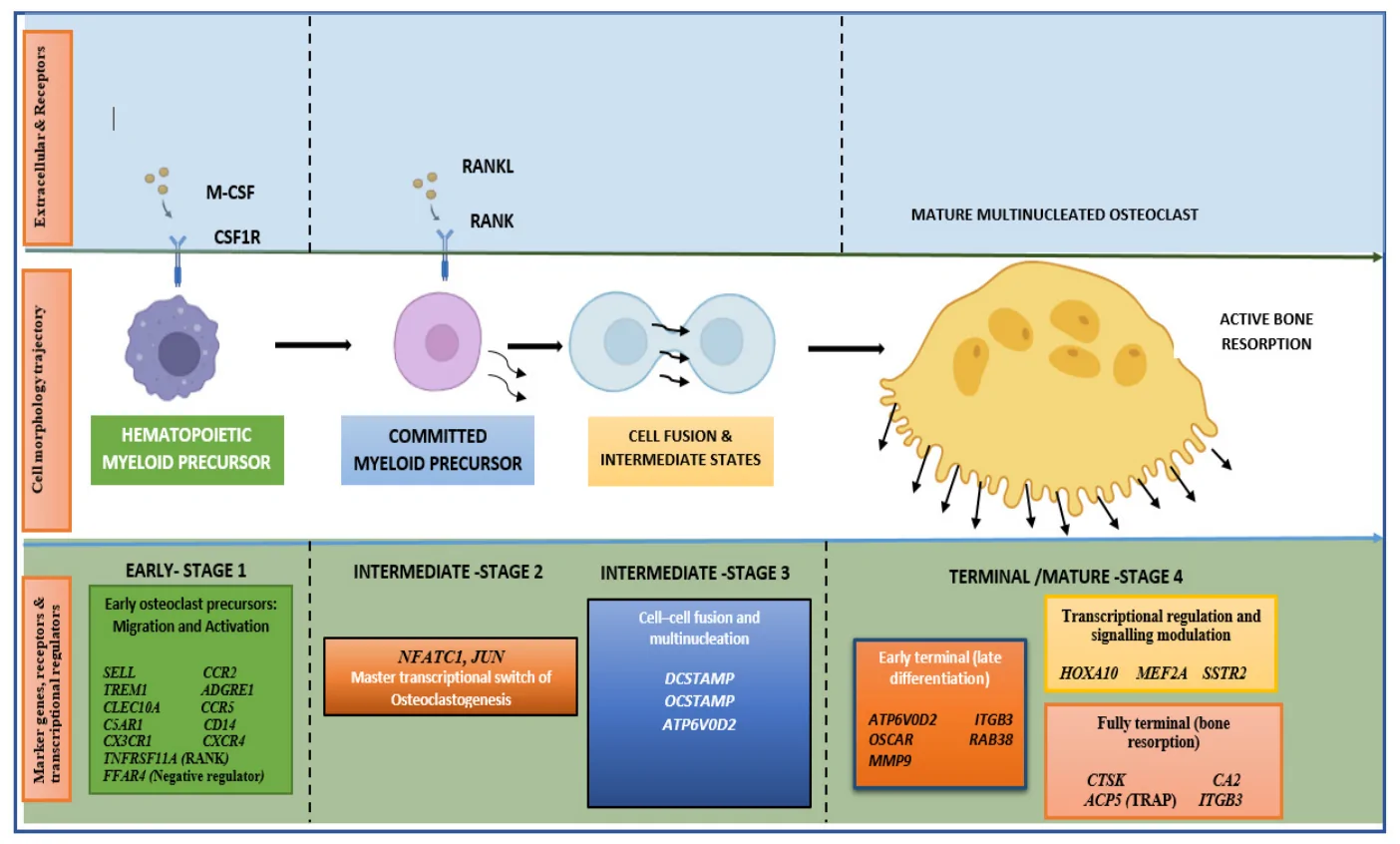
RNA-seq derived four-stage model of osteoclast differentiation and heterogeneity: Schematic illustration mapping the four successive transcriptional stages (early precursor, committed precursor, intermediate, and terminal/mature) governing osteoclastogenesis. The model integrates stage-specific marker gene expression networks and signature transcription factors with the sequential extracellular signaling and morphological shifts that characterize the functional activation of mature multinucleated osteoclasts.

Osteoclasts are multinucleated bone-resorbing cells that play a central role in bone development, remodeling, and repair. They arise from myeloid progenitors that differentiate into monocyte/macrophage precursors and subsequently fuse into mature osteoclasts under the control of M-CSF/CSF1R and RANK/RANKL signaling pathways [79,80]. Key regulatory factors, including NFATc1 and OPG, encoded by NFATC1 and TNFRSF11B, respectively, play important roles in regulating osteoclast differentiation and activity [81,82,83]. Transcriptomic studies using complementary bulk and single-cell approaches have progressively defined the transcriptional programs underlying human osteoclast differentiation, maturation, and cellular heterogeneity (Figure 4).

#### 3.3.1. Human Studies

Morten S. Hansen et al. utilized bulk RNA-seq to examine the global transcriptional reprogramming during differentiation of CD14^+^ human peripheral blood monocytes into osteoclasts [84]. Early osteoclast precursors retained high expression of monocyte-associated genes, including *TREM1*, *SELL*, *CLEC10A*, and *ADGRE1*, which were progressively downregulated during differentiation, whereas canonical osteoclast genes, including *CTSK*, *ACP5*, *DCSTAMP*, *MMP9*, and *CA2*, increased as cells acquired a mature osteoclast phenotype. Consistent with these findings, Sarah Rashid et al. compared differentiated osteoclast-like cells with their precursor PBMCs using bulk RNA-seq [85]. Monocyte-associated genes such as *CCR2*, *CCR5*, and *SELL (*CD62L) were highly expressed in precursor PBMCs, whereas osteoclast-related genes, including *CTSK*, *DCSTAMP*, *ACP5*, *MMP9*, *ATP6V0D2*, and *ITGB3*, were significantly upregulated following differentiation into mature osteoclasts. Notably, both studies identified overlapping transcriptional signatures, with monocyte-associated genes predominating in precursor cells and osteoclast-associated genes becoming enriched during differentiation, providing independent support for a conserved transcriptional transition from precursor to mature osteoclast states.

Building on these findings, scRNA-seq has provided higher-resolution insight into osteoclast heterogeneity and lineage progression. Omata et al. performed an interspecies scRNA-seq analysis of osteoclast-lineage cells derived from human and mouse progenitors and demonstrated that osteoclast cultures are highly heterogeneous, comprising more than ten distinct clusters representing multiple maturation states that coexist simultaneously [86]. In the human dataset, trajectory analysis revealed progression from monocyte–macrophage precursors toward terminally differentiated osteoclasts. Earlier populations retained expression of *CD14*, *CCR2*, *CX3CR1*, *CXCR4*, *TNFRSF11A* (RANK), and *NFATC1*, reflecting progressive lineage commitment, whereas mature osteoclast clusters exhibited high expression of *ACP5*, *CTSK*, *ATP6V0D2*, *DCSTAMP*, *OCSTAMP*, and *OSCAR*. Among these, *ATP6V0D2*, *CTSK*, *ACP5*, and *OCSTAMP* showed particularly strong expression in late, fully differentiated clusters, supporting their association with terminal maturation. *RAB38* was also highly expressed in mature osteoclast clusters, suggesting a potential role in osteoclast function and identifying it as a candidate target for further investigation in bone disease.

A separate human scRNA-seq study using CD14^+^ mononuclear cells isolated from peripheral blood and stimulated with M-CSF and RANKL further resolved the differentiation process into four transcriptionally distinct stages of RANKL-induced osteoclastogenesis [87]. Early osteoclastogenic cells expressed *TXNIP* and *IL7R*, whereas the most mature population showed enrichment of osteoclast differentiation and developmental programs and expressed osteoclast-lineage marker genes including *TYROBP*, *SNX10*, and *FOS*. *JAG1*, encoding the Notch ligand Jagged1, was also highly expressed in the mature population, suggesting stage-specific involvement of Notch signaling during osteoclast maturation. Together with the trajectory identified by Omata et al., these findings support a progressive transition from mononuclear precursors through intermediate states to mature osteoclasts while revealing transcriptionally distinct stages obscured in bulk analyses.

Single-cell profiling has further revealed that osteoclastogenesis includes previously unrecognized intermediate populations with specialized functions. A human cortical bone scRNA-seq study identified a novel early osteoclast-lineage subset termed osteostaticytes (OSCs) [88]. Pseudotime analysis placed OSCs early in the osteoclast-lineage trajectory, preceding preosteoclasts and mature osteoclasts, with high expression of *IDO1*, *CCL3*, and *CCL4*. OSCs showed the highest chemotactic activity among the identified osteoclast-lineage populations, and functional analyses showed they could recruit MSCs, suggesting a role in the reversal phase of bone remodeling and in coupling bone resorption to subsequent bone formation. The trajectory was accompanied by dynamic changes in osteoclast-associated genes, including *CD14*, *ACP5*, and *CTSK*, further supporting distinct transcriptional states as cells progress toward mature osteoclasts. Thus, single-cell approaches not only resolve the progressive maturation of osteoclasts but also reveal specialized subsets with potential roles beyond bone resorption.

The transcriptional diversity of osteoclastogenesis is also influenced by the origin and microenvironment of precursor populations. Fate-mapping combined with scRNA-seq has demonstrated that osteoclasts can arise from distinct precursor populations. Erythromyeloid progenitors were shown to generate a distinct population of osteoclast precursors that contributes to bone homeostasis and repair [89]. This developmental diversity is further expanded by the identification of adipogenic lineage precursors within the bone marrow microenvironment, which promote osteoclast differentiation through the provision of RANKL [90], emphasizing the contribution of both intrinsic lineage origin and niche-derived signals in regulating osteoclast formation.

Beyond identifying cellular states, transcriptomic analyses have provided insight into the regulatory mechanisms governing osteoclast differentiation. Hansen et al. used RNA-seq, pathway analysis, and k-means clustering to show that osteoclastogenesis involves coordinated temporal gene-expression programs rather than isolated changes in individual genes [84]. Early-stage programs were enriched for mitochondrial activity and metabolic reprogramming, consistent with the increased energetic demands of differentiation. In contrast, late-stage upregulated genes, including *CTSK*, *ACP5*, and *DCSTAMP*, were strongly associated with mature osteoclast functions such as ECM degradation, cell–cell fusion, and cytoskeletal remodeling, consistent with the acquisition of bone-resorptive capacity. Network analysis incorporating predicted regulatory interactions of 682 transcription factors further revealed a hierarchical regulatory architecture in which early transcriptional programs initiate intermediate regulatory states that subsequently activate late osteoclast-specific genes. NFATc1 and JUN exhibited increased regulatory activity during differentiation and emerged as central regulatory nodes controlling late-stage osteoclast gene expression. Importantly, regulatory activity was not necessarily reflected by changes in transcription-factor expression, highlighting the value of network-based approaches for identifying functional regulators. Their bulk RNA-seq study also revealed *HOXA10* and *MEF2A* transcription factors, which are predicted to regulate mature osteoclast-associated genes and bone mineral density-related pathways despite minimal changes in their expression levels, suggesting functional regulatory roles independent of transcriptional upregulation [84]. Notably, *HOXA10* is a known regulator of osteoblast differentiation and may contribute to osteoclastogenesis through indirect transcriptional control of Runx2-associated regulatory networks, suggesting broader inter-lineage regulatory roles [91]. Complementing these network-level findings, Tsukasaki et al. used a single-cell transcriptomic dataset to identify CITED2 (CBP/p300-interacting transactivator with Glu/Asp-rich carboxy-terminal domain 2) as an important molecular regulator controlling the decision toward mature osteoclast differentiation [92].

Transcriptomic analyses have also identified G-protein-coupled receptors (GPCRs) as stage-specific modulators of osteoclast differentiation and function [84]. Established regulators included CALCR, LGR4, and GIPR. LGR4, a leucine-rich repeat-containing receptor, modulates RANKL-associated signaling and acts to suppress osteoclast formation and activity. Similarly, GIPR, which mediates signaling of the incretin hormone glucose-dependent insulinotropic polypeptide, inhibits osteoclast differentiation and resorptive capacity. Additional GPCRs with defined signaling roles included *C5AR1*, *SSTR2*, and *FFAR4*. Morten S. Hansen et al. showed that *C5AR1* was predominantly expressed in human osteoclast precursors and declined markedly during differentiation, further supporting its role as an early-stage regulator [84]. Similarly, FFAR4 was identified as an early-stage regulator, with functional studies demonstrating that its activation limits osteoclast differentiation and resorptive activity by suppressing key osteoclastogenic genes, including NFATC1 and DCSTAMP [84]. In contrast, SSTR2 displayed a later functional role, primarily regulating mature osteoclast resorptive activity through suppression of intracellular cAMP signaling without substantially affecting differentiation [84]. Table 3 summarizes the key transcriptomic markers, their functional roles, and the osteoclast differentiation stages identified across these human studies.

Together, these human transcriptomic studies further demonstrate that osteoclast heterogeneity is shaped by precursor origin, bone-marrow niche interactions, and hierarchical transcriptional and signaling networks that collectively regulate osteoclast differentiation and bone-resorptive function.

#### 3.3.2. Animal Studies

Animal models have further elucidated the molecular programs and cellular trajectories underlying osteoclast lineage differentiation. An integrative scRNA-seq and scATAC-seq study in mice identified common monocyte progenitors (cMoPs) and monocytes as critical stages of osteoclast-lineage priming, characterized by coordinated activation of cytoskeletal, immune, and cell-migration programs [93]. At these stages, Irf8, Klf4, and Mafb were expressed as part of the monocyte-associated transcriptional program. IRF8 functioned as a regulatory brake on osteoclastogenesis by maintaining monocyte identity and restricting accessibility of osteoclastogenic loci. Loss of IRF8 increased chromatin accessibility at osteoclast-associated genes, including Nfatc1 and Cebpe, while reducing accessibility of monocyte-associated genes such as Mafb and Klf4, thereby enhancing the osteoclastogenic potential of cMoPs and monocytes. Functional validation further demonstrated that IRF8-deficient monocytes generated increased numbers of TRAP-positive osteoclasts with elevated expression of Nfatc1, Ctsk, Acp5, and Dcstamp. These findings demonstrate that osteoclast lineage commitment is established through coordinated transcriptional and epigenetic remodeling at the early precursor stage.

Extending this concept of precursor heterogeneity, a separate murine scRNA-seq study of bone marrow-derived osteoclast precursors during intermediate RANKL-induced differentiation identified 10 transcriptionally distinct clusters, including early MHC-II^+^ mononuclear precursors, C1q^+^ macrophage-like cells, and Acp5^+^ osteoclast-lineage cells expressing Acp5, Mmp9, and Ctsk [94]. Pseudotime analysis revealed two opposing trajectories originating from MHC-II^+^ precursors, leading either toward C1q^+^ macrophage-like cells or Acp5^+^ osteoclasts, with intermediate clusters representing progressive differentiation states. Along the osteoclast trajectory, Acp5, Ocstamp, Ctsk, and Oscar progressively increased, reflecting acquisition of the osteoclast transcriptional program. Together, these studies show that osteoclastogenesis is not a uniform progression but involves early transcriptional and epigenetic priming followed by heterogeneous precursor states and divergent cell-fate trajectories toward osteoclast populations. A summary of the key transcriptomic markers, their functional roles, and their associated osteoclast differentiation stages identified across these mouse models is provided in Table 4.

### 3.4. Osteoclast Dysregulation in Bone Diseases: Transcriptomic Perspectives

Transcriptomic approaches are widely used in bone biology to investigate gene expression changes associated with various bone diseases and related biological processes. In bone tissue, these analyses capture alterations in gene expression across different conditions, and by comparing healthy and diseased states, they help identify key genes and signaling pathways that are differentially regulated in bone pathology [95]. By profiling gene expression across diverse bone-related cell types such as osteoblasts and osteoclasts, the coordinated interactions and functional roles of these cells in bone regeneration and disease can be better understood [96]. Transcriptomic analyses have revealed that osteoclast gene expression is profoundly altered in pathological conditions associated with excessive bone resorption. Such alterations are evident across diverse disease contexts, including osteoporosis, inflammatory joint disorders, and bone tumors, each characterized by distinct yet overlapping transcriptional programs that drive aberrant osteoclast activity.

#### 3.4.1. Human Studies

Transcriptomic studies across bone and joint diseases have revealed that osteoclast dysregulation involves not only altered expression of osteoclast-associated genes but also changes in lineage states, regulatory networks, and interactions with the surrounding disease microenvironment. In pigmented villonodular synovitis (PVNS)**,** a disorder characterized by synovial proliferation and progressive bone erosion, integrated bulk RNA-seq and microarray analyses by Yang Zhao et al. identified marked upregulation of osteoclast-related genes, including MMP9, MMP11, TNFRSF11A, and OCSTAMP [97]. The elevated expression of these bone-resorption-associated genes was consistent with the extensive bone erosion observed in PVNS, while RT-PCR validation further confirmed increased expression of OCSTAMP, SIGLEC15, and TNFRSF11A compared with osteoarthritis (OA) tissues. Gene Ontology enrichment further implicated osteoclast-related pathways, indicating that osteoclast-associated transcriptional activation contributes to the osteolytic phenotype of PVNS.

Beyond identifying disease-associated osteoclast gene signatures, single-cell approaches have enabled the identification of specific regulatory mechanisms underlying osteoclast differentiation in degenerative bone disease. In human OA subchondral bone, scRNA-seq analysis identified ETS2 as a key transcriptional regulator associated with osteoclast differentiation and predicted CEBPB as a downstream target [98]. Differential expression of ETS2 and CEBPB across osteoclast-lineage populations suggested their involvement in osteoclast differentiation during OA progression. Experimental validation further demonstrated that the ETS2–CEBPB regulatory axis promoted osteoclast differentiation, supporting its role in osteoclastogenesis and OA-associated bone remodeling.

In osteolytic bone tumors, scRNA-seq has further revealed that osteoclast populations are themselves transcriptionally heterogeneous. In giant cell tumor of bone (GCTB), a tumor characterized by extensive osteolysis driven by osteoclast activity, Feng et al. identified three distinct osteoclast populations: progenitor osteoclasts expressing myeloid-associated genes such as C1QA, C1QB, HLA-DRA, CD74, and CD14; mature osteoclasts enriched for classical osteoclast marker genes including ACP5, CTSK, and ATP6V0D2; and dysfunctional osteoclasts displaying reduced expression of osteoclast functional genes [99]. Trajectory analysis indicated progression from progenitor osteoclasts toward either mature or dysfunctional states, demonstrating divergent osteoclast fates within the tumor microenvironment. Mature osteoclasts also showed increased CKLF expression, consistent with enhanced migratory activity during maturation. Ligand–receptor analysis revealed the *TNFRSF11A–TNFSF11* (RANK–RANKL) interaction and identified key signaling pathways regulating osteoclast formation and migration, including RANKL, CD137, PARs, and SEMA3 networks. These findings demonstrate that osteoclasts within GCTB comprise functionally distinct states shaped by tumor-associated signaling.

A similar but distinct pattern of osteoclast heterogeneity has been observed in osteosarcoma (OS). Osteosarcoma, one of the most common primary malignant bone tumors, arises predominantly from mesenchymal tissue and is characterized by aggressive bone destruction. Using scRNA-seq, Sun et al. characterized the cellular composition of OS and identified six osteoclast subgroups, among which a highly proliferative C2 MKI67^+^ osteoclast population was distinguished [100]. This subgroup, characterized by elevated expression of the proliferation-associated marker gene *MKI67*, was positioned early in the differentiation trajectory. Elevated MKI67 expression suggested increased proliferative activity within this population, potentially contributing to enhanced osteoclast-mediated bone resorption and OS progression. Pathway analysis identified activation of the amyloid precursor protein (APP) signaling pathway, with APP–CD74 interactions implicated in osteoclast formation and activity within the C2 MKI67^+^ population. Regulatory network analysis highlighted *PPARG* as the most active transcription factor within the C2 MKI67^+^ osteoclast population. *PPARG*, which encodes the nuclear receptor PPARG-γ, promotes osteoclast differentiation and activity, enhancing bone resorption and disrupting bone homeostasis. Collectively, these findings suggest that MKI67-driven proliferation, APP-mediated signaling, and PPARG-dependent transcriptional regulation converge to enhance osteoclast function in OS.

Transcriptomic studies of metastatic disease have further extended this concept by demonstrating direct tumor–osteoclast interactions. In a human scRNA-seq study of lung adenocarcinoma (LUAD), primary tumor and bone-metastatic tissues from six patients were analyzed to investigate mechanisms underlying osteolytic metastasis [101]. They identified reduced *NPC2* expression in tumor cells associated with bone metastasis. The analysis also revealed interactions between *NPC2*-low tumor cells and osteoclast-like cells, suggesting that *NPC2* loss in tumor cells may contribute to osteoclast activation and osteolytic disease progression. Subsequent experimental validation showed that *NPC2* downregulation in LUAD cells promoted osteoclast differentiation and maturation, whereas *NPC2* overexpression reduced these effects, supporting a role for NPC2-mediated tumor–osteoclast crosstalk in osteolytic bone metastasis.

Collectively, these studies demonstrate that transcriptomic approaches have substantially advanced the understanding of disease-specific osteoclast dysregulation by revealing enhanced osteoclastogenic programs, altered regulatory pathways, distinct osteoclast states, and disease-associated cellular interactions, thereby providing mechanistic insight into pathological bone resorption and identifying potential therapeutic targets.

#### 3.4.2. Animal Studies

Animal-model studies further show that disease states are associated with transcriptional alterations in osteoclast-lineage precursor populations that can enhance or suppress their osteoclastogenic potential. Activated osteoclasts contribute substantially to inflammatory bone destruction in arthritis and other conditions associated with excessive bone loss. In a murine collagen-induced arthritis model, transcriptomic profiling of CCR2^high^ and CCR2^low^ osteoclast progenitors revealed distinct molecular states [102]. CCR2^high^ progenitors showed stronger activation of osteoclastogenic, chemokine, and inflammatory programs, indicating greater capacity for osteoclast formation together with increased responsiveness to signals that promote migration toward inflamed tissues. In contrast, CCR2^low^ progenitors exhibited characteristics of a less differentiated, bone-marrow-resident population associated with basal osteoclast production under normal conditions. These findings suggest that arthritis alters the osteoclast progenitor compartment by expanding a highly osteoclastogenic and inflammation-responsive CCR2^high^ population, potentially contributing to localized pathological bone resorption.

Extending beyond defined osteoclast progenitors, scRNA-seq has identified distinct macrophage subpopulations in inflamed joint tissues that exhibit osteoclastogenic potential and contribute to pathological osteoclast formation. In a study by Hasegawa et al. [103], macrophages isolated from the inflamed synovial “bare area” of arthritic mice were classified as a distinct population, termed arthritis-associated osteoclastogenic macrophages (AtoMs). Trajectory analysis revealed that the transcription factor FoxM1 enhances the differentiation potential of both murine and human AtoMs into osteoclasts, highlighting FoxM1 as a potential therapeutic target for modulating osteoclast-mediated bone destruction in rheumatoid arthritis.

Beyond inflammatory arthritis, transcriptomic profiling has also revealed disease-specific alterations in osteoclast precursor function under metabolic stress. In type 2 diabetic mice, scRNA-seq demonstrated suppression of major osteoclastogenic pathways and reduced AP-1 (Fos/Jun) activity, particularly *Fosb*, among osteoclast precursors [104]. The identification of a Cd36^+^ monocyte/macrophage population with reduced osteoclast differentiation potential further indicated that type 2 diabetes can impair osteoclastogenesis at the precursor level, providing transcriptomic evidence that metabolic disease alters the bone immune microenvironment and osteoclast differentiation.

## 4. Integrative Transcriptomic Approaches in Bone Biology: Challenges and Emerging Multi-Modal Solutions

### 4.1. Challenges in Bulk and Single-Cell RNA Sequencing for Bone Research

Bulk RNA-seq quantifies transcript abundance from a pooled cell population, producing an averaged gene expression profile, whereas scRNA-seq resolves transcriptomes at single-cell resolution, enabling identification of cell-to-cell variability and distinct cellular subpopulations [105]. The integration of scRNA-seq and bulk RNA-seq has increasingly been used to investigate bone-related diseases in greater depth within transcriptomic and multi-omics frameworks, enabling both cell-type-specific and tissue-level insights.

Among these applications, postmenopausal osteoporosis (PMOP) has attracted considerable attention owing to its high prevalence and its association with estrogenic deficiency-induced disruption of bone remodeling. PMOP is a skeletal disorder that occurs in women after menopause and is characterized by progressive bone loss resulting from reduced estrogen levels. In this context, Fuzhu Tan integrated scRNA-seq and bulk RNA-seq data to explore the role of lactylation-related genes (LRGs) in PMOP [106]. Their analytical pipeline combined differential expression analysis, Mendelian randomization, and machine learning to develop a diagnostic model for disease classification. The study also showed that cell populations stratified by high and low LRG expression exhibited distinct transcriptional profiles. Moreover, the study identified several lactylation-associated genes, including *CSRP2*, *FUBP1*, *S100A9*, and *ARHGEF10*, as potential biomarkers involved in PMOP pathogenesis.

Although the integration of bulk and scRNA-seq has significantly advanced transcriptomic profiling in bone biology, these approaches remain constrained by the loss of spatial organization and the lack of multi-layered molecular resolution in complex tissue systems. To address these challenges, spatial transcriptomics and multi-omics technologies have emerged as complementary frameworks for achieving a more comprehensive understanding of skeletal biology.

### 4.2. Beyond Bulk and Single-Cell RNA Sequencing: Spatial Transcriptomics and Multi-Omics Approaches

#### 4.2.1. Spatial Transcriptomics in Bone Biology

Spatial transcriptomics has emerged as a powerful approach for mapping gene expression while maintaining spatial context within intact tissues [107,108]. By preserving tissue architecture, this technology enables high-resolution localization of distinct cellular states within their native microenvironment and facilitates the investigation of spatially organized cell–cell interactions that are not accessible through dissociative sequencing approaches [109,110]. Consequently, spatial transcriptomics provides important insights into how tissue organization and cellular neighborhoods influence physiological and pathological processes within the skeletal system [111]. In applied settings, spatial transcriptomics has been used to analyze human biopsy specimens and map localized morphological and gene expression changes in bone disorders, including osteoarthritis, primary bone tumors, and metastatic disease [112].

Beyond pathological conditions, spatial transcriptomics has significantly advanced the understanding of bone regeneration and repair mechanisms. Wang et al. applied spatial transcriptomic profiling to periosteal tissue following fracture to map cellular dynamics during healing [113]. Their analysis revealed a transition from mesenchymal progenitor cells (MPCs) towards a regenerative MPC (rMPC) state, which appears to orchestrate early repair events, including the recruitment of macrophages to the fracture site. A subset of rMPCs exhibited transcriptional features resembling proliferative progenitor cells (PPCs), with increased expression of genes such as *Acta2*, *Lrrc15*, and *Tagln*, particularly during the early stages of fracture healing. These PPC-like cells were found to play a crucial role in stem cell activation and subsequent differentiation during bone regeneration. Complementary scRNA-seq analysis further highlighted Bmp2 activity within the rMPC and osteogenic populations during early and intermediate healing phases, reinforcing its role as a key mediator of bone regeneration. In addition, regulatory network analysis identified transcription factors including *Meox2*, *En1*, and *Foxp1*, as critical regulators of rMPC function, thereby contributing to the spatial and temporal coordination of fracture repair.

#### 4.2.2. Bone Sample Preparation for Spatial Transcriptomics

Bone tissue presents unique challenges for spatial transcriptomic analysis because its mineralized ECM can complicate tissue sectioning, molecular accessibility, and RNA preservation [114]. Therefore, careful optimization of tissue preparation is essential for obtaining reliable spatial transcriptomic data.

One of the major technical challenges in spatial transcriptomic analysis of bone is the removal of mineral components through decalcification. Inappropriate decalcification conditions may reduce RNA integrity, resulting in lower unique molecular identifier (UMI) and gene counts and affecting the reliability of spatial transcriptomic datasets [113]. Recent methodological work has demonstrated that bone decalcification can be achieved while preserving RNA and tissue architecture when the processing conditions are appropriately optimized [113]. Decalcification methods can be broadly classified into acid-based and chelating approaches. Strong acids, such as nitric and hydrochloric acid, rapidly remove mineral components but may compromise tissue integrity. Weak acids, including formic acid, provide a slower alternative and are commonly used in routine tissue processing [115]. In contrast, chelating agents, particularly ethylenediaminetetraacetic acid (EDTA), remove calcium more gradually and are generally preferred for research applications because they better preserve tissue morphology and molecular integrity. Compared with hydrochloric- or formic-acid treatment, EDTA-based decalcification better preserves nucleic acid quality and supports downstream molecular analyses, including probe-based techniques such as fluorescence in situ hybridization (FISH). Therefore, EDTA-based protocols are generally more suitable for transcriptomic studies of bone tissue [116,117].

Beyond decalcification, tissue fixation and processing also influence the quality of spatial transcriptomic analysis. Two commonly used approaches are fresh-frozen (FF) processing and formalin-fixed, paraffin-embedded (FFPE) processing. In FFPE processing, tissue is fixed in formalin, dehydrated, embedded in paraffin, sectioned, and subsequently deparaffinized for spatial transcriptomic analysis. This approach provides excellent preservation of tissue morphology and permits long-term storage at room temperature. However, formalin-induced crosslinking and chemical modification of RNA can reduce RNA integrity and accessibility. In contrast, FF processing generally preserves RNA with less fragmentation and is compatible with a broader range of spatial transcriptomic platforms without additional RNA recovery steps. However, FF samples require continuous cold-chain storage and may be more susceptible to sectioning-related artifacts, such as tissue tearing. Unlike FFPE blocks, they also cannot be stored indefinitely at room temperature [118]. Thus, neither approach is universally optimal, and the choice should be guided by the study objectives, with FFPE favoring morphological preservation and long-term storage, while FF offers advantages for RNA preservation and transcript detection.

The choice of spatial platform should subsequently be matched to the biological objective of the study, tissue preparation, required spatial resolution, transcript coverage, and detection sensitivity [119]. Spatial transcriptomic technologies can broadly be divided into imaging-based and sequencing-based approaches [108,120]. Imaging-based methods are largely based on single-molecule fluorescence in situ hybridization (smFISH), in which multiple probe sets are designed to bind specific RNA transcripts within tissue sections [121]. In highly multiplexed approaches, numerous primary probes hybridize to their corresponding target transcripts, followed by fluorophore-labeled secondary probes that bind the primary probes, enabling simultaneous visualization of thousands of RNA targets. Some platforms can detect up to approximately 6000 transcripts in a single experiment [119]. Major imaging-based platforms include Xenium, MERSCOPE, and CosMx, which provide high spatial resolution for examining cellular organization and localized gene expression. In contrast to imaging-based approaches, sequencing-based technologies couple spatially barcoded arrays with next-generation sequencing to determine both transcript abundance and spatial location within tissue [122]. In these approaches, poly(dT) sequences incorporated into spatially barcoded capture probes bind polyadenylated mRNA, after which the spatial barcodes are retained during cDNA synthesis. Following library preparation and sequencing, the resulting transcripts can therefore be assigned to their corresponding locations based on their spatial barcodes [123]. Major sequencing-based platforms, including 10× Visium, Visium HD, Visium CytAssist, Stereo-seq, and Slide-seq, enable transcriptome-wide spatial profiling at different spatial resolutions [119]. For example, conventional Visium, Visium HD, and Stereo-seq differ substantially in the feature size of their capture arrays, with reported dimensions of approximately 55 μm, 2 μm, and 0.22 μm, respectively. Visium CytAssist additionally facilitates whole-transcriptome profiling across preserved tissue sections, including FFPE samples, whereas higher-resolution approaches such as Slide-seqV2 and Stereo-seq may be advantageous when fine cellular architecture or specific cell–cell interactions need to be resolved [119]. Therefore, the selection of a spatial transcriptomic platform should ultimately be guided by the experimental requirements and the biological question being addressed.

Overall, successful spatial transcriptomic analysis of bone requires careful tissue preparation, including appropriate fixation, optimized decalcification, and assessment of RNA and tissue quality before selecting a compatible spatial platform. Such optimization is essential to preserve transcript detection and spatial information and to obtain reliable resolution of cellular populations and their spatial relationships within bone.

#### 4.2.3. Integration of Spatial Transcriptomics with RNA Sequencing in Skeletal Biology

To achieve higher-resolution mapping of bone tissue architecture and cellular interactions, spatial transcriptomics is increasingly integrated with scRNA-seq approaches. A recent landmark study by Lin et al. demonstrated the power of integrating spatial transcriptomics with scRNA-seq to resolve the spatial organization of the human bone marrow microenvironment [42]. Lin et al. generated an integrative high-resolution spatial map of human bone and bone marrow by combining spatial transcriptomics with scRNA-seq from human femoral tissue. Their integrative analysis revealed a specialized bone formation niche enriched in osteoblastic lineage (OstLin) cells and fibroblasts, predominantly located around trabecular bone, characterized by upregulation of *COL1A1*, *SPP1*, *MMP9*, and *SPARC*, and associated with bone formation, maintenance, and repair. A spatial gradient extending from trabecular bone showed systematic variation in gene expression, cellular composition, and pathway activity, with *RUNX2* expression and OstLin abundance decreasing with distance, whereas immune cells, particularly B cells, showed the opposite distribution. Pathway analyses showed decreasing PI3K, TGFβ, and VEGF signaling with increasing distance, while NFKB and TNFα pathways initially increased before moderately declining, reflecting spatially regulated inflammatory activity.

Spatial transcriptomics has also advanced the understanding of tumor heterogeneity and stromal organization in skeletal malignancies. Extending this framework to pathological contexts, Zhang et al. applied a multi-platform approach combining scRNA-seq, spatial transcriptomics, bulk RNA-seq, and multiplexed immunofluorescence to investigate cancer-associated fibroblasts in chordoma [124]. scRNA-seq identified a distinct CAF subpopulation enriched in endoplasmic reticulum stress-related pathways and characterized by high expression of hypoxia-associated genes, including *HIF1A*, *VEGFR*, and *BAG3*, suggesting a role in tumor progression. Spatial transcriptomic analysis further confirmed the presence of this CAF subtype within tumor tissues and demonstrated its close spatial proximity to tumor cells. Similarly Bo-Wen Zheng et al. integrated scRNA-seq, bulk RNA-seq, and spatial transcriptomics to characterize cancer-associated fibroblasts in chordoma [125]. Their analysis identified an inflammatory CAF (iCAF) subpopulation marked by elevated expression of inflammatory genes, suggesting a role in promoting chordoma initiation and progression. Integration with spatial transcriptomic data further confirmed the presence of this iCAF subset within tumor tissues and showed that these cells were spatially localized at a distance from tumor cells.

Similarly, integrative transcriptomic approaches have been applied to investigate bone metastases in prostate cancer. Ihle et al. utilized RNA sequencing in combination with Digital Spatial Profiling (DSP) to analyze decalcified FFPE bone tissue samples containing metastatic prostate cancer lesions [126]. Their analysis revealed distinct transcriptional differences between osteolytic and osteoblastic metastases. Lytic lesions showed increased expression of genes such as *TREM2*, *CYBB*, *PTGER4*, *WNT5A*, and *S100A9* and were enriched for PI3K–AKT signaling, whereas blastic lesions were characterized by higher expression of *SHC2*, *NEIL1*, *ITGA2*, *LAMC2*, and *MMP7*, along with enrichment of JAK–STAT pathway genes.

While recent single-cell studies have significantly advanced our understanding of osteoclast heterogeneity in osteosarcoma, the tumor microenvironment comprises multiple interacting cellular compartments that collectively drive disease progression. In particular, endothelial cells (ECs) play essential roles in tumor angiogenesis, progression, and metastasis. The application of scRNA-seq has enabled detailed characterization of EC heterogeneity, revealing transcriptionally distinct subpopulations with specialized functions [127]. For instance, He et al. demonstrated functional diversity among ECs in lung metastatic lesions of osteosarcoma, highlighting their contribution to metastatic progression [128]. More recently, Tang et al. integrated scRNA-seq with spatial transcriptomics to further resolve EC heterogeneity and spatial organization within osteosarcoma [129]. Their analysis identified multiple EC clusters, including a tip-like EC subpopulation characterized by elevated expression of *IGFBP3*, *MCAM*, *THY1*, *INSR*, *MMP2*, and *COL4A1*. Notably, this tip-like EC population was enriched in the primary tumor group, suggesting a potential role in tumor initiation and metastatic dissemination. Among these markers, melanoma cell adhesion molecule (MCAM) was prominently upregulated in tip-like ECs compared to other endothelial subtypes. Spatial transcriptomic analysis revealed that MCAM-positive ECs were localized in close proximity to osteoblast-like tumor cells within metastatic lymph node tissue, indicating potential cell–cell interactions that may facilitate tumor progression.

#### 4.2.4. Multi-Omics Approaches in Bone Biology

Despite advances in spatial transcriptomics, a comprehensive understanding of skeletal biology requires integrating multiple molecular layers, as single-omics approaches offer limited mechanistic resolution. Bone physiology and pathology are regulated through interconnected genomic, epigenomic, transcriptomic, proteomic, and metabolic processes that cannot be fully resolved using single-omics approaches [130]. Multi-omics technologies address this limitation by integrating complementary molecular datasets, thereby enabling systems-level characterization of the regulatory networks governing bone development, remodeling, regeneration and disease pathogenesis [131]. Consequently, integrative multi-omics approaches also contribute to personalized medicine by enabling the identification of patient-specific molecular signatures and distinguishing distinct pathological mechanisms underlying bone loss and related musculoskeletal disorders [132,133]. Genomics, transcriptomics, proteomics, and metabolomics enable large-scale analysis of DNA, RNA, proteins, and metabolites, providing integrated insight into bone regeneration under both normal and pathological conditions [134].

In osteoporosis, estrogen deficiency disrupts skeletal homeostasis, leading to an imbalance between bone formation and resorption. While therapeutic strategies have traditionally focused on limiting osteoclast-mediated bone resorption, multi-omics evidence suggests that additional cellular and molecular mechanisms contribute to disease progression. For example, Feng et al. investigated BMSCs derived from ovariectomized (OVX) rats, an established model of postmenopausal estrogen deficiency-induced bone loss, using integrated proteomic and transcriptomic analysis [135]. Their results demonstrated altered osteogenic and adipogenic differentiation potential, accompanied by significant changes in genes and proteins enriched in ECM–receptor interaction, PI3K–Akt signaling, and Wnt signaling pathways. In particular, dysregulation of ECM components, including collagen proteins such as COL1A1 and COL1A2, was associated with impaired organization of the bone microenvironment and altered bone turnover. Collectively, these findings indicate that estrogen deficiency-associated osteoporosis involves not only increased bone resorption but also impaired osteogenic potential of BMSCs and disruption of ECM-mediated regulatory networks.

Traditional single-layer analyses that focus solely on mRNA or protein expression are often insufficient to fully capture the complexity of gene regulation in musculoskeletal disorders. To address this limitation, Chen J. applied an integrated transcriptomic and proteomic approach to investigate the molecular relationship between sarcopenia and osteoporosis [136]. By analyzing bone and muscle tissue samples from patients with osteoporosis and osteosarcopenia, the study identified several genes and proteins that were differentially expressed at both the transcriptional and protein levels, including *PDIA5*, *TUBB1*, *CYFIP2*, *MYH7*, and *NCAM1*, which showed significant alterations at both transcriptional and protein levels. Among these, *NCAM1* and *PDIA5* emerged as potential biomarkers associated with disease progression and severity. Furthermore, pathway analysis highlighted osteoclast differentiation and NF-κB signaling as key mechanisms involved in osteosarcopenia, indicating possible therapeutic targets for future interventions.

Similarly, Tan Z. et al. employed an integrated multi-omics strategy combining proteomics, transcriptomics, and scRNA-seq to investigate type XV osteogenesis imperfecta, a rare autosomal recessive skeletal disorder associated with *WNT1* mutations [137]. Their findings demonstrated markedly reduced SOST expression in type XV patients relative to individuals with *COL1A1* variants. Furthermore, single-cell transcriptomic analysis revealed impaired osteogenic cell populations characterized by diminished *WNT1* expression, enrichment of CXCL12^+^ progenitor cells, reduced mature osteocytes, and the emergence of adipogenic-like cell subsets, highlighting disrupted skeletal cell differentiation and altered bone microenvironmental composition.

Furthermore, Zhang B et al. combined single-cell sequencing with transcriptomic profiling to investigate immune-related mechanisms involved in osteoporosis [138]. Using approaches including intercellular communication mapping, pseudotime trajectory analysis, and high-dimensional Weighted Gene Co-expression Network Analysis (hdWGCNA), the study identified specific immune cell populations and regulatory genes associated with disease progression. Their findings highlighted an increased presence of neutrophils in osteoporotic samples, suggesting an important contribution of innate immune responses to osteoporosis development. In addition, the hub genes *DND1*, *HIRA*, *SH3GLB2*, and *F7* were proposed as potential mediators of inflammatory and metabolic dysregulation in the bone microenvironment, suggesting their potential as diagnostic biomarkers and therapeutic targets. In another study, an integrative multi-omics approach was employed to investigate shared molecular mechanisms between osteoporosis and type 1 diabetes [139]. This strategy enabled the computational identification of *CPNE1* and *FRAT2* as candidate genes potentially involved in both conditions. These findings suggest that these genes may participate in convergent pathways linking bone metabolic dysfunction with systemic metabolic regulation.

Integrating multiple omics layers enables a more comprehensive understanding of disease mechanisms by linking genomic variation with downstream transcriptional changes. In a study by Eisfeldt J et al., multi-omics analysis of patient clinical data identified a reciprocal translocation between chromosomes 17 and 19 in individuals with osteoporosis [140]. This structural variant was detected using long-read genome sequencing (Nanopore lrGS). Integrated analyses implicated *MINK1* as a potential monogenic disease gene associated with osteoporosis. Furthermore, transcriptomic profiling demonstrated reduced *MINK1* expression in patient-derived osteoporotic samples, supporting its functional relevance in disease pathogenesis.

Multi-omics approaches, driven by rapid technological advances, improved computational tools, and the increasing availability of commercial platforms, have enabled the extension of multi-omics analyses to the single-cell level in bone biology. A key example is that the integration of scRNA-seq with single-nucleus ATAC sequencing (snATAC-seq) represents a multi-omics approach that links gene expression profiles with chromatin accessibility landscapes [131,141]. This integrated strategy supports the characterization of diverse bone-resident and bone marrow-derived cell populations based on gene expression patterns, while also allowing inference of underlying regulatory programs and dynamic processes involved in bone remodeling and disease progression.

Spatial transcriptomics and multi-omics approaches have further advanced transcriptomic research by introducing spatial and systems-level dimensions to molecular profiling. Together, these approaches have strengthened bone research by enabling more detailed analysis of individual cell activity while also maintaining spatial context, helping to clarify how different bone-related cells interact within specific regions and contributing to a deeper understanding of bone maintenance and disorders such as osteoporosis [131].

## 5. Future Directions in Bone Transcriptomic Research

Recent advances in bulk RNA-seq, scRNA-seq, spatial transcriptomics, and integrative multi-omics have substantially expanded our understanding of bone biology by revealing cellular heterogeneity, lineage dynamics, and disease-associated molecular pathways. Despite these achievements, several challenges remain that must be addressed to fully realize the translational potential of these technologies. A major limitation of current transcriptomic studies is the lack of systematic functional validation of the genes, pathways, and regulatory networks identified. While high-throughput sequencing approaches have generated extensive datasets and uncovered numerous candidate molecular regulators of bone formation, resorption, and disease progression, many findings remain largely correlative. Future studies should therefore prioritize experimental validation using in vitro and in vivo models, gene perturbation strategies, and lineage-specific functional analyses to establish causal relationships and clarify the biological significance of transcriptomic discoveries.

Variation in cellular sources and experimental populations is an important consideration when comparing transcriptomic studies addressing the same skeletal lineage. Studies may analyze whole bone, bone marrow, PBMCs, mixed stromal or non-hematopoietic populations, lineage-specific primary cells, or progenitor populations differentiated in vitro. Whole-tissue and mixed-population approaches may capture transcripts from multiple stromal, hematopoietic, immune, and skeletal populations, whereas lineage-focused approaches provide more direct insight into cell-specific transcriptional programs. Although mixed-population studies are valuable for investigating the bone microenvironment and cellular interactions, differences in sample composition and cellular or differentiation states can influence the molecular signatures detected and contribute to variation in reported genes and pathways across studies. These differences should therefore be considered when interpreting and comparing findings within the same skeletal lineage.

In addition to this limitation, the interpretation of bone-related omics data is further constrained by incomplete integration across multiple molecular layers. Although integration of transcriptomic data with individual omics platforms has already yielded important insights into bone biology, future progress will depend on the simultaneous integration of multi-layered molecular datasets, encompassing genomic, epigenomic, proteomic, metabolomic, and spatial information. Such multi-omics frameworks, coupled with advances in computational and systems biology approaches, are expected to more effectively resolve the complex regulatory architecture governing skeletal homeostasis and disease than single- or dual-omics strategies. This holistic, systems-level integration holds substantial promise for refining mechanistic understanding of bone physiology and pathogenesis.

Despite these advances, significant computational and technical challenges continue to limit the full application of high-dimensional omics technologies in bone research. Single-cell datasets are inherently sparse and high-dimensional and are further affected by batch-to-batch variation and differences in analytical pipelines. Similarly, spatial transcriptomic approaches are constrained by limited spatial resolution, complex data structures, and variability in experimental workflows, which may impact reproducibility and biological interpretation. Furthermore, integrating heterogeneous multi-modal datasets remains challenging due to differences in scale, resolution, and pre-processing requirements across platforms. These challenges are further compounded by ethical considerations related to the governance, sharing, and clinical use of patient-derived genomic data, which may affect large-scale translational implementation [142,143,144,145]. Collectively, addressing these limitations through improved experimental validation, advanced integrative computational frameworks, and enhanced spatial and multi-omics technologies will be essential for transforming high-throughput molecular profiling into a robust mechanistic and translational toolset for skeletal biology and bone disease research.

## 6. Conclusions

Transcriptomic technologies have transformed the study of bone biology by enabling comprehensive characterization of skeletal cell populations, their differentiation trajectories, and the molecular mechanisms governing bone formation, remodeling, and pathology. The integration of bulk RNA-seq, scRNA-seq, spatial transcriptomics, and multi-omics platforms has shifted skeletal research from single-dimensional gene expression analyses to a systems-level understanding of tissue homeostasis. These technologies have uncovered previously unrecognized stromal, osteogenic, and osteoclastogenic subpopulations, clarified lineage relationships, and identified key regulatory genes involved in skeletal homeostasis and disease.

While existing literature often focuses on isolated cellular lineages or individual sequencing technologies, this review addresses a distinct niche by providing a stage-resolved, cross-study synthesis of both bone-forming and bone-resorbing lineages. By integrating evidence distributed across multiple independent studies and transcriptomic platforms, this review compiles key regulatory markers and stage-associated signatures for BMSCs, osteoblasts, and osteoclasts into a unified framework. A central asset of this review is the extensive cross-study tabulated evidence, which allows gene expression signatures reported across diverse experimental models, species, and sequencing platforms to be evaluated side by side. Rather than relying on findings from single isolated datasets, our multi-study integration confirms the recurrent detection of core transcriptional signatures across independent literature. Specifically, across progressive stages of osteoblastogenesis, the consistent identification of marker genes such as *RUNX2*, *SP7*, *BGLAP*, *MMP13*, *SPP1*, *COL1A1*, *IBSP*, and *DMP1* across multiple transcriptomic datasets provides robust cross-validation of their stage-specific regulatory roles. Similarly, in osteoclastogenesis, cross-dataset profiling confirms the recurrent expression of key functional marker genes including *CTSK*, *ACP5*, *DCSTAMP*, *MMP9*, and *NFATC1* across distinct maturation phases. By consolidating and analyzing these distributed transcriptomic findings into a unified matrix, this review provides validated, evidence-based marker sets while highlighting context-dependent variations across experimental settings. By combining data from multiple studies into a single clear framework, this review highlights reliable gene markers for each developmental stage while showing how they vary across different experimental conditions.

Importantly, by presenting the transcriptional programs of normal lineage progression alongside pathological alterations, this review provides a novel comparative baseline for understanding skeletal disease. Comparing standard differentiation trajectories with disease-associated transcriptomic profiles, particularly in conditions involving dysregulated osteoclastogenesis and excessive bone resorption, allows alterations in stage-associated gene expression and regulatory programs to be identified more clearly. These transcriptomic approaches also extend beyond basic biology by providing insights into disease-associated gene dysregulation in inflammatory bone disorders and bone tumors, supporting the identification of lineage-specific biomarkers and potential molecular targets. Such findings may contribute to the development of strategies to modulate osteogenic and osteoclastogenic activity and may support future approaches to early diagnosis, patient stratification, and personalized treatment.

Collectively, these complementary transcriptomic and multi-omics approaches offer high translational value for precision medicine. As spatial and multi-omics technologies continue to evolve, integrating these high-resolution molecular datasets with clinical parameters will accelerate biomarker discovery, refine patient stratification, and guide targeted therapeutic strategies for metabolic, inflammatory, and neoplastic bone disorders.

## Figures and Tables

**Table 1:** Transcriptomic markers, regulatory factors, and functional signatures associated with distinct stages and cellular populations of human osteoblast differentiation.

Genes (Key Markers/Transcription Factors/Regulators)	Functional Role	Differentiation Stage/Cell Population	Species	Sample/Cell Source	Cellular Composition/Study Population	Reference
*LEPR*, *FOXC1*, *FOS*, *JUN*, *JUNB*, *JUND*	Early progenitor activation and AP-1–mediated transcriptional priming.	Early osteoblast progenitors.	Human	Primary osteoblasts isolated from the femoral head.	Osteoblast-lineage focused	[73]
*LEPR*, *VCAM1*, *CD99*, *APP*	Transition of mesenchymal stromal cells toward preosteoblast lineage commitment.	Preosteoblast stage.	Human	Primary osteoblasts isolated from the femoral head.	Osteoblast-lineage focused	[73]
*IGFBP2*, *LOXL1*, *NR4A1*, *NR4A2*, *ATF3*, *NAMPT*	Transcriptional reprogramming and metabolic adaptation during the intermediate stage.	Intermediate osteoblast clusters.	Human	Primary osteoblasts isolated from the femoral head.	Osteoblast-lineage focused	[73]
*BGLAP*, *SPP1*, *IBSP*	Terminal osteoblast maturation and ECM mineralization.	Mature osteoblasts.	Human	Primary osteoblasts isolated from the femoral head.	Osteoblast-lineage focused	[73]
*ALPL*, *COL1A1*, *RUNX2*, *SPARC*, *OMD*, *OGN*	Stage-dependent activation of the osteogenic program, involving osteoblast differentiation, ECM production, collagen organization, and mineralization.	Early-to-mature osteogenic differentiation stage.	Human	Human iPSC-derived MSCs, differentiated in vitro into preosteoblasts and osteoblasts.	Osteoblast-lineage focused; iPSC-derived osteogenic populations	[74]
*KLF16*	Negative regulator of osteogenic differentiation.	Across MSC → preosteoblast → osteoblast trajectory.	Human	Human iPSC-derived MSCs, differentiated in vitro into preosteoblasts and osteoblasts.	Osteoblast-lineage focused; iPSC-derived osteogenic populations	[74]
IBSP, BGLAP, SPP1 *WIF1*, *SFRP4*	Late osteoblast maturation. Specifically WIF1, SFRP4 reported as inhibitors of osteogenic differentiation.	BMSC → preosteoblast → osteoblast differentiation. trajectory	Human	Human BMSCs isolated from bone marrow of the femoral diaphysis; primary osteoblasts isolated from the femoral head.	Osteoblast-lineage focused	[72]
ADIPOQ	Adipogenic differentiation potential.	Adipogenic-primed BMSC marker/Early stage.	Human	Human BMSCs isolated from bone marrow of the femoral diaphysis; primary osteoblasts isolated from the femoral head.	Osteoblast-lineage focused	[72]
*ADAM28*, *FZD1*	Associated with the osteogenic differentiation trajectory.	Early Osteogenic BMSC population.	Human	Human BMSCs isolated from bone marrow of the femoral diaphysis; primary osteoblasts isolated from the femoral head.	Osteoblast-lineage focused	[72]
*NR4A1*, *NR4A2*	Associated with osteoblast differentiation and inhibition of osteoclast recruitment.	Preosteoblast/Intermediate stage.	Human	Human BMSCs isolated from bone marrow of the femoral diaphysis; primary osteoblasts isolated from the femoral head.	Osteoblast-lineage focused	[72]
*ATF3*, *CCL2*, *CXCL2*, *IRF1*, *IER3*	Associated with a less differentiated/differentiation-arrested state and osteoclast recruitment.	Preosteoblast/Intermediate stage.	Human	Human BMSCs isolated from bone marrow of the femoral diaphysis; primary osteoblasts isolated from the femoral head.	Osteoblast-lineage focused	[72]
FOS, FOSB, JUNB, EGR1	Stress-responsive transcriptional program associated with adipogenic progenitor identity.	Early stromal cluster subsets considered as HAGEPs (highly adipocytic gene-expressing progenitors).	Human	Human bone marrow stromal cells from healthy donors.	Non-hematopoietic bone marrow stromal cells; mixed stromal population; in vitro tri-lineage differentiation	[71]
RUNX1, CDH11, EBF1, EBF3	Osteogenic commitment and progression toward the osteoblast lineage.	Preosteoblast stage.	Human	Human bone marrow stromal cells from healthy donors.	Non-hematopoietic bone-marrow stromal cells; mixed stromal population; in vitro tri-lineage differentiation	[71]
*BGLAP*, *CHAD*, *SPP1*, *RUNX2*,*CDH11*, *CDH2*, *IBSP*, *SPARC*	Osteochondrogenic differentiation.	Osteochondrogenic progenitors.	Human	Human bone marrow stromal cells from healthy donors.	Non-hematopoietic bone-marrow stromal cells; mixed stromal population; in vitro tri-lineage differentiation	[71]
*RBFOX2*	Gene encoding RBP involved in regulation of osteogenic differentiation; highly expressed in osteoblasts and experimentally shown to promote osteogenic differentiation.	Terminal/Mature osteoblast stage.	Human	Publicly available transcriptomic datasets analyzed through GENEVESTIGATOR: osteoblasts (*n* = 4, GSE12264).	Osteoblast-lineage focused	[75]

**Table 2:** Transcriptomic markers, regulatory factors, and functional signatures associated with distinct stages of osteoblast differentiation and cellular heterogeneity in mouse models.

Genes (Key Markers/Transcription Factors/Regulators)	Functional Role	Differentiation Stage/Cell Population	Species	Sample/Cell Source	Cellular Composition/Study Population	Reference
Bmp2, *Runx2*, Atf3, Atf4, Ddit3	Early osteogenic lineage commitment accompanied by stress-responsive transcriptional activation.	Early osteogenic stage.	Mouse	Primary calvarial osteoblasts (pCOBs) isolated from newborn C57BL/6 wild-type mice, differentiated in- vitro to mineralization.	Osteoblast-lineage focused; primary osteoblast population.	[76]
Col1a1, Actn4, Aspn, Dcn, Lum.	ECM synthesis, collagen deposition, and matrix organization.	Intermediate Osteogenic stage.	Mouse	Primary calvarial osteoblasts (pCOBs) isolated from newborn C57BL/6 wild-type mice, differentiated in- vitro to mineralization.	Osteoblast-lineage focused; primary osteoblast population.	[76]
*Mmp13*	Matrix remodeling.	Early and late intermediate stage.	Mouse	Primary calvarial osteoblasts (pCOBs) isolated from newborn C57BL/6 wild-type mice, differentiated in- vitro to mineralization.	Osteoblast-lineage focused; primary osteoblast population.	[76]
*Bglap*, *Sost*, *Ibsp*, *Dmp1*, *Spp1*	Terminal osteoblast maturation and ECM mineralization, characterized by ossification and bone formation processes.	Late osteoblast maturation/mineralization stage.	Mouse	Primary calvarial osteoblasts (pCOBs) isolated from newborn C57BL/6 wild-type mice, differentiated in- vitro to mineralization.	Osteoblast-lineage focused; primary osteoblast population.	[76]
*Runx2*, *Sp7*, *Mmp13*, *Postn*, *Spp1*	Osteogenic lineage commitment and early differentiation, characterized by activation of type I collagen-based ECM formation.	Preosteoblast stage.	Mouse	BMSCs isolated from the femur and tibia of 6–8-week-old C57BL/6 (CD45.2) mice.	Non-hematopoietic bone and bone marrow cells comprising a mixed stromal population of MSCs and osteolineage cells.	[70]
*Alpl*, *Tnfrsf11b* (OPG), *Col1a1*	Asynchronous maturation with heterogeneous osteogenic marker expression and active collagen matrix production.	Intermediate osteoblast stage.	Mouse	BMSCs isolated from the femur and tibia of 6–8-week-old C57BL/6 (CD45.2) mice.	Non-hematopoietic bone and bone marrow cells comprising a mixed stromal population of MSCs and osteolineage cells.	[70]
*Bglap2*, *Col1a1*, *Sparc*, *Ifitm5*	ECM organization and mineralization.	Mature osteoblast stage.	Mouse	BMSCs isolated from the femur and tibia of 6–8-week-old C57BL/6 (CD45.2) mice.	Non-hematopoietic bone and bone marrow cells comprising a mixed stromal population of MSCs and osteolineage cells.	[70]
*Bglap*, *Ibsp*, *Dmp1*	Canonical osteoblast maturation toward osteocytogenesis.	Mature osteoblast stage.	Mouse	Venus^+^ primary osteoblasts isolated from newborn mouse calvariae.	Osteoblast-lineage focused.	[77]
*Dmp1*, *Phex*, *Cd34*, *Cxcl12*, *Kitl*, *Angpt1*	Bone marrow niche support and progenitor cell maintenance.	Mature osteoblast stage.	Mouse	Venus^+^ primary osteoblasts isolated from newborn mouse calvariae.	Osteoblast-lineage focused.	[77]
*Ereg*, *Efcab2*	Promote ALP activity and ECM mineralization.	Mature osteoblast stage.	Mouse	MC3T3-E1 mouse osteoblastic cell line, in vitro osteoblastic differentiation.	Osteoblast-lineage focused.	[78]
*Hrc*, *Gli1*, *Ifitm5*	Negative regulators of osteoblast differentiation and maturation.	Mature osteoblast stage.	Mouse	MC3T3-E1 mouse osteoblastic cell line, in vitro osteoblastic differentiation.	Osteoblast-lineage focused.	[78]

**Table 3:** Transcriptomic markers, regulatory factors, and functional signatures associated with distinct stages and cellular populations of human osteoclast differentiation.

Genes (Key Markers/Transcription Factors/Regulators)	Functional Role	Differentiation Stage/Cell Population	Species	Sample/Cell Source	Cellular Composition/Study Population	Reference
*TREM1*, *SELL*, *CLEC10A*, *ADGRE1*	Monocyte-associated program before osteoclast commitment.	Early osteoclast precursor stage	Human	Primary human osteoclasts differentiated from human CD14+ monocytes	Osteoclast-lineage focused	[84]
*CTSK*, *ACP5*, *DCSTAMP*, *MMP9*, *CA2*	Bone resorption, matrix degradation, acidification, and cell fusion.	Mature osteoclast stage	Human	Primary human osteoclasts differentiated from human CD14+ monocytes	Osteoclast-lineage focused	[84]
*CCR2*, *CCR5*, *SELL* (CD62L)	Monocyte-associated marker genes expressed prior to lineage commitment.	Early osteoclast precursor stage	Human	PBMC-derived osteoclast-like cells differentiated in vitro	Hematopoietic population; mixed PBMC-derived precursor population	[85]
*CTSK*, *DCSTAMP*, *ACP5*, *MMP9*, *ATP6V0D2*, *ITGB3*	Coordinated bone-resorbing program involving fusion, adhesion, acidification, and matrix degradation enabling osteoclast maturation.	Mature osteoclasts	Human	PBMC-derived osteoclast-like cells differentiated in vitro	Hematopoietic population; mixed PBMC-derived precursor population	[85]
*ACP5*, *CTSK*, *ATP6V0D2*, *OCSTAMP*, *DCSTAMP*, *OSCAR*	Activated bone-resorbing program with osteoclast maturation, acidification, matrix degradation, and resorption pit formation.	Late/terminal differentiation stage (mature osteoclasts)	Human	Human PBMC- differentiated in vitro with M-CSF and RANKL	Osteoclast-lineage focused; heterogeneous osteoclast-lineage population	[86]
*CD14*, *CCR2*, *CX3CR1*, *CXCR4*	Immune-like precursor program controlling monocyte identity, chemotaxis, and tissue homing before osteoclast commitment.	Monocyte/macrophage precursor population	Human	Human PBMC- differentiated in vitro with M-CSF and RANKL	Osteoclast-lineage focused; heterogeneous osteoclast-lineage population	[86]
*TNFRSF11A* (RANK), *NFATC1*	Initiation of osteoclast differentiation program via RANK–NFATc1 axis controlling lineage commitment and transcriptional activation.	Early lineage commitment stage	Human	Human PBMC- differentiated in vitro with M-CSF and RANKL	Osteoclast-lineage focused; heterogeneous osteoclast-lineage population	[86]
*RAB38*	Regulate osteoclast lysosome-related organelle (LRO) biogenesis and trafficking for resorptive compartment organization.	Mature osteoclasts	Human	Human PBMC- differentiated in vitro with M-CSF and RANKL	Osteoclast-lineage focused; heterogeneous osteoclast-lineage population	[86]
*C5AR1*	Positive regulation of early osteoclast precursor differentiation and commitment.	Early osteoclast precursor stage	Human	Primary human osteoclasts differentiated from human CD14+ monocytes	Osteoclast-lineage focused	[84]
*FFAR4*	Negative regulation of osteoclast maturation and resorptive activity through suppression of *NFATC1* and *DCSTAMP*.	Early-to-intermediate osteoclast differentiation	Human	Primary human osteoclasts differentiated from human CD14+ monocytes	Osteoclast-lineage focused	[84]
*NFATC1*, *JUN*	Key transcription factors activating late-stage osteoclast-specific gene expression.	Intermediate-to-differentiated osteoclast trajectory	Human	Primary human osteoclasts differentiated from human CD14+ monocytes	Osteoclast-lineage focused	[84]
*HOXA10*, *MEF2A*	Predicted regulators of mature osteoclast transcriptional programs and bone remodeling via RUNX2 networks.	Late/terminal osteoclast differentiation	Human	Primary human osteoclasts differentiated from human CD14+ monocytes	Osteoclast-lineage focused	[84]
*SSTR2*	Stage-specific modulation of mature osteoclast bone-resorbing activity via cAMP signaling without affecting differentiation.	Mature osteoclast population	Human	Primary human osteoclasts differentiated from human CD14+ monocytes	Osteoclast-lineage focused	[84]
*TXNIP*, *IL7R*	Early osteoclastogenic/progenitor program.	Early osteoclastogenic progenitor stage	Human	CD14^+^CD16^−^ monocytes isolated from human PBMCs and differentiated in vitro with M-CSF + RANKL	Osteoclast-lineage focused; CD14^+^ monocyte-derived heterogeneous differentiation population	[87]
*TYROBP*, *SNX10*, *FOS*	Osteoclast differentiation and lineage development.	Later/more mature osteoclast-lineage stage	Human	CD14^+^CD16^−^ monocytes isolated from human PBMCs and differentiated in vitro with M-CSF + RANKL	Osteoclast-lineage focused; CD14^+^ monocyte-derived heterogeneous differentiation population	[87]
IDO1, CCL3, CCL4	Osteostaticytes represent an osteoclast-lineage subset associated with coupling bone resorption to subsequent bone formation.	Early osteoclast-lineage subset (OSCs)	Human	Huma cortical bone tissue from femur/tibia	Mixed bone-cell population; stromal, hematopoietic, vascular, and bone-lineage populations	[88]

**Table 4:** Transcriptomic markers, regulatory factors, and functional signatures associated with distinct stages of osteoclast differentiation and cellular heterogeneity in mouse models.

Genes (Key Markers/Transcription Factors/Regulators)	Functional Role	Differentiation Stage/Cell Population	Species	Sample/Cell Source	Cellular Composition/Study Population	Reference
MHC-II^+^	Early osteoclast precursor population that serves as a branching point for cell-fate decisions	Early osteoclast precursor stage	MOUSE	Bone marrow cells from femur and tibia of ~12-week-old C57BL/6JRj mice, induced with M-CSF and RANKL.	Osteoclast-lineage focused; heterogeneous osteoclast differentiation state	[94]
C1q^+^	Macrophage-like population representing the alternative macrophage differentiation trajectory arising from MHC-II^+^ precursors	Macrophage-directed differentiation stage	MOUSE	Bone marrow cells from femur and tibia of ~12-week-old C57BL/6JRj mice, induced with M-CSF and RANKL.	Osteoclast-lineage focused; heterogeneous osteoclast differentiation state	[94]
*Acp5*, *Ocstamp*, *Ctsk*, *Oscar*, *Mmp9*	Progressive increase along the osteoclast trajectory, indicating progressive commitment toward the osteoclast phenotype	Intermediate-to-differentiated osteoclast trajectory	MOUSE	Bone marrow cells from femur and tibia of ~12-week-old C57BL/6JRj mice, induced with M-CSF and RANKL.	Osteoclast-lineage focused; heterogeneous osteoclast differentiation state	[94]
*Irf8*, *Mafb*, *Klf4*	Maintenance of monocyte identity and restriction of osteoclastogenic programming	Common monocyte progenitors (cMoPs)/early monocyte stage	MOUSE	Primary bone marrow cells were isolated from the femurs and tibias of Irf8 WT and Irf8 cKO mice.	Hematopoietic; mixed bone-marrow hematopoietic populations spanning HSPCs, cMoPs, and monocytes	[93]

## Data Availability

No new data were created or analyzed in this study. Data sharing is not applicable to this article.
